# Applying machine learning to assist in the morphometric assessment of brain arteriolosclerosis through automation

**DOI:** 10.17879/freeneuropathology-2025-6387

**Published:** 2025-06-02

**Authors:** Jerry J. Lou, Peter Chang, Kiana D. Nava, Chanon Chantaduly, Hsin-Pei Wang, William H. Yong, Viharkumar Patel, Ajinkya J. Chaudhari, La Rissa Vasquez, Edwin Monuki, Elizabeth Head, Harry V. Vinters, Shino Magaki, Danielle J. Harvey, Chen-Nee Chuah, Charles S. DeCarli, Christopher K. Williams, Michael Keiser, Brittany N. Dugger

**Affiliations:** 1 Department of Pathology and Laboratory Medicine, School of Medicine, University of California Irvine, Irvine, USA; 2 Department of Radiological Sciences, Center for Artificial Intelligence in Diagnostic Medicine, School of Medicine, University of California Irvine, Orange, USA; 3 Department of Pathology and Laboratory Medicine, School of Medicine, University of California Davis, Sacramento, USA; 4 Department of Electrical and Computer Engineering, University of California Davis, Davis, USA; 5 Department of Pathology and Laboratory Medicine and Department of Neurology, David Geffen School of Medicine, University of California Los Angeles, Los Angeles, USA; 6 Department of Pathology and Laboratory Medicine, David Geffen School of Medicine, University of California Los Angeles, Los Angeles, USA; 7 Department of Public Health Sciences, School of Medicine, University of California Davis, Davis, USA; 8 Department of Neurology, School of Medicine, University of California Davis, Sacramento, USA; 9 Department of Pharmaceutical Chemistry, Department of Bioengineering and Therapeutic Sciences, Institute for Neurodegenerative Diseases; Bakar Computational Health Sciences Institute, University of California San Francisco, San Francisco, USA

**Keywords:** Machine learning, Artificial intelligence, Neuropathology, Arteriolosclerosis, Blood vessel, Morphometry

## Abstract

Objective quantification of brain arteriolosclerosis remains an area of ongoing refinement in neuropathology, with current methods primarily utilizing semi-quantitative scales completed through manual histological examination. These approaches offer modest inter-rater reliability and do not provide precise quantitative metrics. To address this gap, we present a prototype end-to-end machine learning (ML)-based algorithm, Arteriolosclerosis Segmentation (ArtSeg), followed by Vascular Morphometry (VasMorph) – to assist persons in the morphometric analysis of arteriolosclerotic vessels on whole slide images (WSIs). We digitized hematoxylin and eosin-stained glass slides (13 participants, total 42 WSIs) of human brain frontal or occipital lobe cortical and/or periventricular white matter collected from three brain banks (University of California, Davis, Irvine, and Los Angeles Alzheimer’s Disease Research Centers). ArtSeg comprises three ML models for blood vessel detection, arteriolosclerosis classification, and segmentation of arteriolosclerotic vessel walls and lumens. For blood vessel detection, ArtSeg achieved area under the receiver operating characteristic curve (AUC-ROC) values of 0.79 (internal hold-out testing) and 0.77 (external testing), Dice scores of 0.56 (internal hold-out) and 0.74 (external), and Hausdorff distances of 2.53 (internal hold-out) and 2.15 (external). Arteriolosclerosis classification demonstrated accuracies of 0.94 (mean, 3-fold cross-validation), 0.86 (internal hold-out), and 0.77 (external), alongside AUC-ROC values of 0.69 (mean, 3-fold cross-validation), 0.87 (internal hold-out), and 0.83 (external). For arteriolosclerotic vessel segmentation, ArtSeg yielded Dice scores of 0.68 (mean, 3-fold cross-validation), 0.73 (internal hold-out), and 0.71 (external); Hausdorff distances of 7.63 (mean, 3-fold cross-validation), 6.93 (internal hold-out), and 7.80 (external); and AUC-ROC values of 0.90 (mean, 3-fold cross-validation), 0.92 (internal hold-out), and 0.87 (external). VasMorph successfully derived sclerotic indices, vessel wall thicknesses, and vessel wall to lumen area ratios from ArtSeg-segmented vessels, producing results comparable to expert assessment. This integrated approach shows promise as an assistive tool to enhance current neuropathological evaluation of brain arteriolosclerosis, offering potential for improved inter-rater reliability and quantification.

## Introduction

 The study of vascular morphology pertains to several neurological disorders, including dementia, stroke, and cerebral vasculopathies. Vascular contributions to cognitive impairment and dementia have emerged from decades of study, including the Honolulu Asia Aging Study,^1^ the Rotterdam Study,^2^ and the Religious Orders Study and Memory and Aging Project.^3,4^ Vascular abnormalities can encompass numerous entities, including brain arteriolosclerosis. Arteriolosclerosis can be associated with an increased likelihood of microinfarcts^5^ and subsequently dementia, including Alzheimer disease (AD).^6,7^

 Currently, the guidelines used in Vascular Cognitive Impairment Neuropathology Guidelines (VCING)^8^ and the National Alzheimer’s Coordinating Center Neuropathology Data Set^9^ for assessing the severity of vascular pathology such as brain arteriolosclerosis (B-ASC) consists of a semi-quantitative scale that divides vascular pathologic change categorically into “none”, “mild”, “moderate”, and “severe”.^10^ According to VCING, occipital cortex white matter may be the optimal brain region for brain arteriolosclerosis assessment in terms of reliability and association with cognitive status.^8^ The semi-quantitative B-ASC scale showed “moderate” inter-rater reliability (Gwet’s AC2 coefficient 0.52) in the VCING study.^8^ Alternative methods have been proposed for the analysis of arteriolosclerosis, including quantitative assessments such as the sclerotic index [1-(internal diameter / external diameter)].^11,12^ However, implementing these methods on a large scale may not be feasible. Scalable means for deep phenotyping of vascular pathology to assess morphological features such as vessel shape, wall thickness, and/or degree of hyaline change are needed. The demand for reproducible and high-resolution quantitative metrics motivates the development of robust computational methods to segment vascular structures. 

 Machine learning (ML) is a promising computational paradigm capable of delivering expert-level performance in complex visual recognition tasks, including the classification of amyloid plaques,^13^ neurofibrillary tangles,^14^ and glial tauopathies.^15^ Additional work has correlated machine learning generated quantitative findings with clinical, demographic, and pathological metrics.^16^ These ML models trained on whole slide image (WSI) datasets recognize features and patterns specific to target objects and demonstrate remarkable adaptability to variations generated by disparate institutional procedures in the production of WSIs. 

In addition to classification, ML models can perform semantic segmentation by which a class label is assigned to each pixel in an image, generating collections of pixels called segmentation masks that form distinct objects within an image. These masks may be used for downstream analysis of the shape, diameter, and/or other features of the segmented object. Generally, classification requires recognition of only a minimum number of object-specific features to categorize images into the correct class, whereas segmentation demands recognition of all object-specific features to classify all pixels belonging to the target.

 Non-ML methods for segmentation and quantification of blood vessels have been explored, however, these methods were not extensively evaluated and compared to expert annotations.^17^ Prior works have shown that ML models can segment non-arteriolosclerotic blood vessels in neoplastic tissue.^18^ We build upon these prior efforts and hypothesize that ML models could (1) detect and localize blood vessels in brain tissue, (2) classify arteriolosclerosis, (3) segment the walls and lumens of arteriolosclerotic vessels, and (4) facilitate morphometric analysis of vascular structure. As object occlusion has been shown to reduce model performance,^23^ we additionally hypothesized centering image patches onto the object of interest, the blood vessel in this study, mitigates occlusion and improves downstream segmentation performance. 

 In this pilot study, we present a prototype end-to-end ML-based algorithm – Arteriolosclerosis Segmentation (ArtSeg), followed by Vascular Morphometry (VasMorph) – to assist persons in the morphometric analysis of arteriolosclerotic vessels on WSIs. While previous software for manual measurement of sclerotic index on digital histology images (VasCalc)^24^ has been described, VasMorph represents an automated method to measure sclerotic index, vessel wall thickness, and vessel wall to lumen area ratio from ArtSeg-segmented vessels. Furthermore, we describe a novel custom recursive wrapper algorithm – Object of interest Recursive Centering Algorithm (ORCA) – that can flexibly interface with any segmentation ML model to recursively generate patches centered on an object of interest. 

We evaluated three ML models within ArtSeg that show promising performance for the detection of blood vessels, recognition of arteriolosclerosis, and segmentation of arteriolosclerotic vessel walls and lumens. To aid in reproducibility and open science, we provide our code, training, and testing data, and image processing methodology (see Data Availability and Code Availability). To the best of our knowledge, this study constitutes the first demonstration of an open-source means for ML-based morphometric analysis of arteriolosclerotic blood vessels in digital histology images of human brain.

## Methods

### Participant consent and ethics compliance

Our investigation used de-identified human post-mortem tissues, which do not qualify as Human Subjects under federal law (45 CFR 46, Protection of Human Subjects). The University of California, Davis (UCD) Alzheimer’s Disease Research Center (ADRC), University of California Irvine (UCI) ADRC, and the former University of California Los Angeles (UCLA) ADRC programs obtained signed informed consent from all participants or legal representatives during the life of the participant. Procedures were completed in accordance with the ethical standards of the Helsinki Declaration. Operations of the UCD, UCI, and former UCLA ADRC were approved by the Institutional Review Board (IRB) of UCD, UCI, and UCLA, respectively. All data were de-identified and shared through a randomly generated pseudo-identification number. The de-identified data does not contain personal health information such as addresses, phone numbers, names, date of birth, or social security numbers.

### Participant selection and case cohort

**Figure 1: Overview of the process for sampling and digitization of slides:**
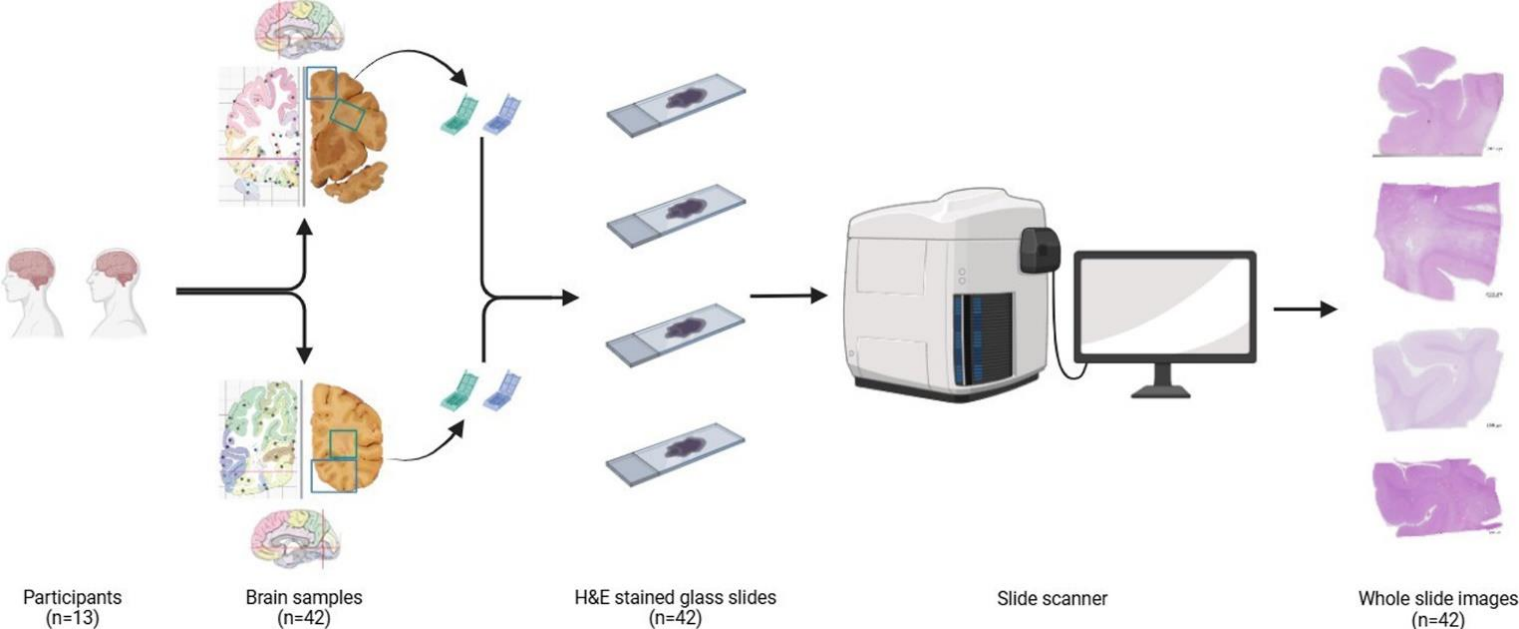
Samples were retrieved from the cortical and/or periventricular white matter regions of the frontal and occipital lobes from 13 participants. These samples were formalin-fixed paraffin-embedded and processed into glass slides, which subsequently were digitized into WSIs by a slide scanner.

 All brain samples were retrieved from archives of UCD ADRC, UCI ADRC, and the former UCLA ADRC. Thirteen participants and 42 WSIs were included through three stages of selection. In the first stage, we chose 1 participant with mild arteriolosclerosis (global score) and 1 with severe arteriolosclerosis (global score) from UCI ADRC, each of whom had frontal and occipital lobe H&E-stained slides. We also chose 2 participants with mild arteriolosclerosis (global score) and 2 with severe arteriolosclerosis (global score) from UCD ADRC, each of whom had frontal, frontal-periventricular white matter, occipital, and occipital-periventricular white matter H&E-stained slides. In the second stage, we added 6 additional participants from UCD ADRC (2 with no arteriosclerosis, 1 with mild, 2 with moderate, and 1 with severe arteriosclerosis, global score) to expand the distribution of arteriosclerosis and increase the sample size. In the third stage, we added 1 participant from the former UCLA ADRC to create an external test set. Cortical and subcortical (periventricular white matter) regions were chosen due to implications in possible vascular contributions to cognitive impairment and dementia.^7^ Samples studied consisted of 5–7 μm formalin-fixed paraffin-embedded sections of the frontal and occipital lobes mounted on glass slides and stained with hematoxylin and eosin (**Figure 1**). UCD ADRC digitized slides with a Zeiss Axioscan scanning at 0.220 μm/pixel and x20 magnification. The former UCLA ADRC utilized an Aperio CS2 with the same parameters as UCD ADRC. UCI ADRC digitized slides with an Aperio Versa 200 scanner at 0.137 μm/pixel with a 40x objective. In total, our study included 8 male and 5 female participants from 52 to 89+ years of age (mean 81, median 86). See **Supplementary Table 1** for detailed specifications and participant demographics of each WSI utilized and **Supplementary Figure 1** for example WSIs from respective brain regions. 

### Inclusion criteria for the selection of vessels

 Only vessels (1) within white matter and (2) cut in cross-section with the entire circumference of the vessel wall within view were included for training and evaluation of the ML algorithm. Because arteriolosclerosis is most common in cortical white matter, to optimize workflow, we chose to focus on only blood vessels located within white matter. We included the occipital lobe because the occipital cortex white matter may be the optimal brain region for brain arteriolosclerosis assessment in terms of reliability and association with cognitive status, according to VCING,^8^ Cross-sectional views of vessels were selected as the purpose of the ML algorithm is to automate the calculation of the sclerotic index,^11,12^ vessel wall thickness, and vessel wall to lumen area ratio, which require measurements of the internal and external radius or diameter. 

### Dataset

 All UCI and UCLA WSIs were stored in SVS file format. All UCD WSIs were stored in CZI file format. Due to the enormous gigapixel size of WSIs, it is generally computationally inefficient or intractable to input the entire WSI directly into ML algorithms for training or inference, and WSIs are typically divided into thousands of smaller image tiles. We performed WSI tiling utilizing the open-source libraries OpenSlide^25^ and czifile,^26^ generating (512 x 512) pixel tiles. For arteriolosclerosis classification and segmentation, we divided our dataset (11 participants, 34 WSIs) into three subsets: a training and validation subset, an internal hold-out test set, and an external test set (**Supplementary Table 2**). The training and validation set consisted of 28 WSIs from 8 participants (26 WSIs from 7 UCD participants and 2 WSIs from 1 UCI participant). We performed a 3-fold cross-validation procedure with on average 19 training and 9 validation WSIs (**Supplementary Tables 3 and 4**) and reported the mean performance metrics of the three folds. The internal hold-out testing subset consisted of 4 WSIs from 2 participants (2 WSIs from 1 UCD participant and 2 WSIs from 1 UCI participant; **Supplementary Table 5**). The external testing subset consisted of 2 WSIs from 1 UCLA participant (**Supplementary Table 5**). For vessel detection, the training and validation subsets had an additional 8 WSIs from an additional 2 UCD participants (**Supplementary Table 6**), who did not have arteriolosclerosis and therefore were not included in the dataset for arteriolosclerosis classification and segmentation. 

### Human annotations

 All annotators (VP, WY, JJL, HPW, KN) were blinded to WSI specifications and participant clinical information during annotation. VP and WY are attending neuropathologists; JJL is a neuropathology fellow; HPW was a junior specialist; and KN was an undergraduate student. All annotations for the training and validation subsets, as well as the internal hold-out test subset, were completed by JJL, HPW, and/or KN. All annotations for the external test subset were completed by VP. Arteriolosclerosis was defined using criteria proposed by Skrobot et al.^8,10^ Segmentation masks for vessel walls and lumens were created by JJL, HPW, KN, and VP using ImageJ^27^ (detailed annotation protocol available in **Supplementary Document 1**). WY annotated vessel wall thicknesses (see section Vascular Morphometry or VasMorph) using ImageJ^27^ (detailed annotation protocol available in **Supplementary Document 2**). 

### Data augmentation

 For all training tiles, color augmentation developed by Tellez et al.^28^ to simulate the spectrum of hues generated by different staining methods was performed through the open-source library HistomicsTK.^29^ Tensorflow’s Keras application programming interface^30^ produced morphological augmentations including random flip, random translation, and random rotation. Gray scale augmentation through the open-source library OpenCV^31^ assisted further in reducing neural network dependence on color. For datasets with class imbalance, minority class oversampling was performed. The optimized color-to-gray augmentation ratio for arteriolosclerotic vessel segmentation was 5:1. For arteriolosclerosis classification, the optimized ratio was 2:1. 

### Overview of end-to-end machine learning-based pipeline

Our end-to-end ML-based pipeline consists of an ML component (**Phase 1**) and a post-ML quantification module (**Phase 2**). The ML component, Arteriolosclerosis Segmentation (ArtSeg) receives WSIs as input and outputs segmentations of arteriolosclerotic blood vessel walls and lumens, which are in turn input into the post-ML module Vascular Morphometry (VasMorph), which outputs quantitative metrics for the sclerotic index, vessel wall thickness, and vessel wall to lumen area ratio.

#### Phase 1: Arteriolosclerosis Segmentation (ArtSeg)

**Figure 2: ArtSeg overview:**
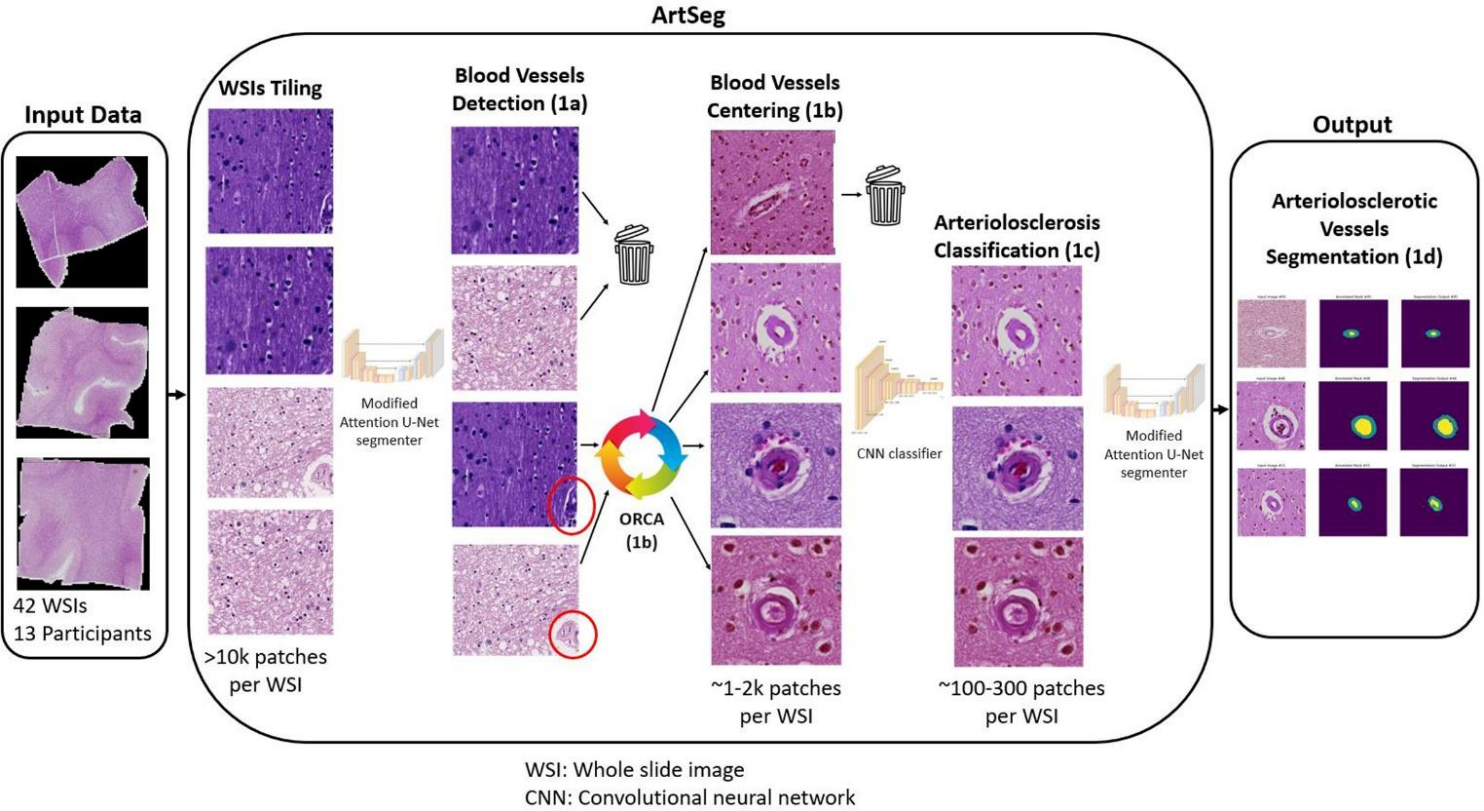
The ML pipeline received WSIs of H&E-stained cortical and/or periventricular white matter brain tissue as input. Each WSI was tiled into tens of thousands of (512 x 512) pixel image tiles. (**Phase 1a**) The blood vessel detection ML model sorted tiles into those with blood vessels and those without. (**Phase 1b**) Object of interest: Recursive Centering Algorithm (ORCA) (see **Figure 3**) generated new tiles centered onto the detected blood vessels. (**Phase 1c**) An arteriolosclerosis classification model separated tiles with centered blood vessels into those with arteriolosclerosis and those without. (**Phase 1d**) A modified Attention U-Net segmented the arteriolosclerotic vessel walls and lumens to produce the final output. All models within ArtSeg take advantage of fixed ImageNet pretrained parameters from Google’s EfficientV2L to extract low-level features before learning vessel-specific features de novo.

ArtSeg comprises four algorithms that complete four sequential steps (**Figure 2**). After WSI tiling, the first step (**Phase 1a**) is to detect blood vessels and keep tiles that contain a blood vessel and discard those that do not. The second step (**Phase 1b**) is to recursively shift tiles until the detected blood vessel appears at the center of the tile. The third step (**Phase 1c**) is to keep tiles that contain a blood vessel with arteriolosclerosis and discard those that do not. The fourth step (**Phase 1d**) is to segment the walls and lumens of blood vessels with arteriolosclerosis.

#### Phase 1a: Blood vessel detection

 The blood vessel detection neural network consisted of an Attention U-net architecture^32^ with an encoder composed of an EfficientNetV2L^33^ backbone with five semi-trainable convolution layers followed by two fully trainable convolution layers and a decoder composed of seven trainable convolution layers generated through the concatenation of a 2D transpose convolution of the prior layer and an attention gate^32^ that filters features propagated from the skip connections. 

To evaluate the classification performance of the vessel detection model for separating patches with blood vessels from those without, the segmentation output of the model was converted to confidence scores by: (1) obtaining the softmax probability per pixel for background (class = 0) and vessel (class = 1), (2) for each pixel where the softmax probability for the vessel class is greater than that for background, saving the vessel softmax probabilities into one set, and (3) calculating the mean of the set (**Supplementary Figure 2**).

#### Phase 1b: Blood vessel centering

**Figure 3: Object of interest Recursive Centering Algorithm (ORCA):**
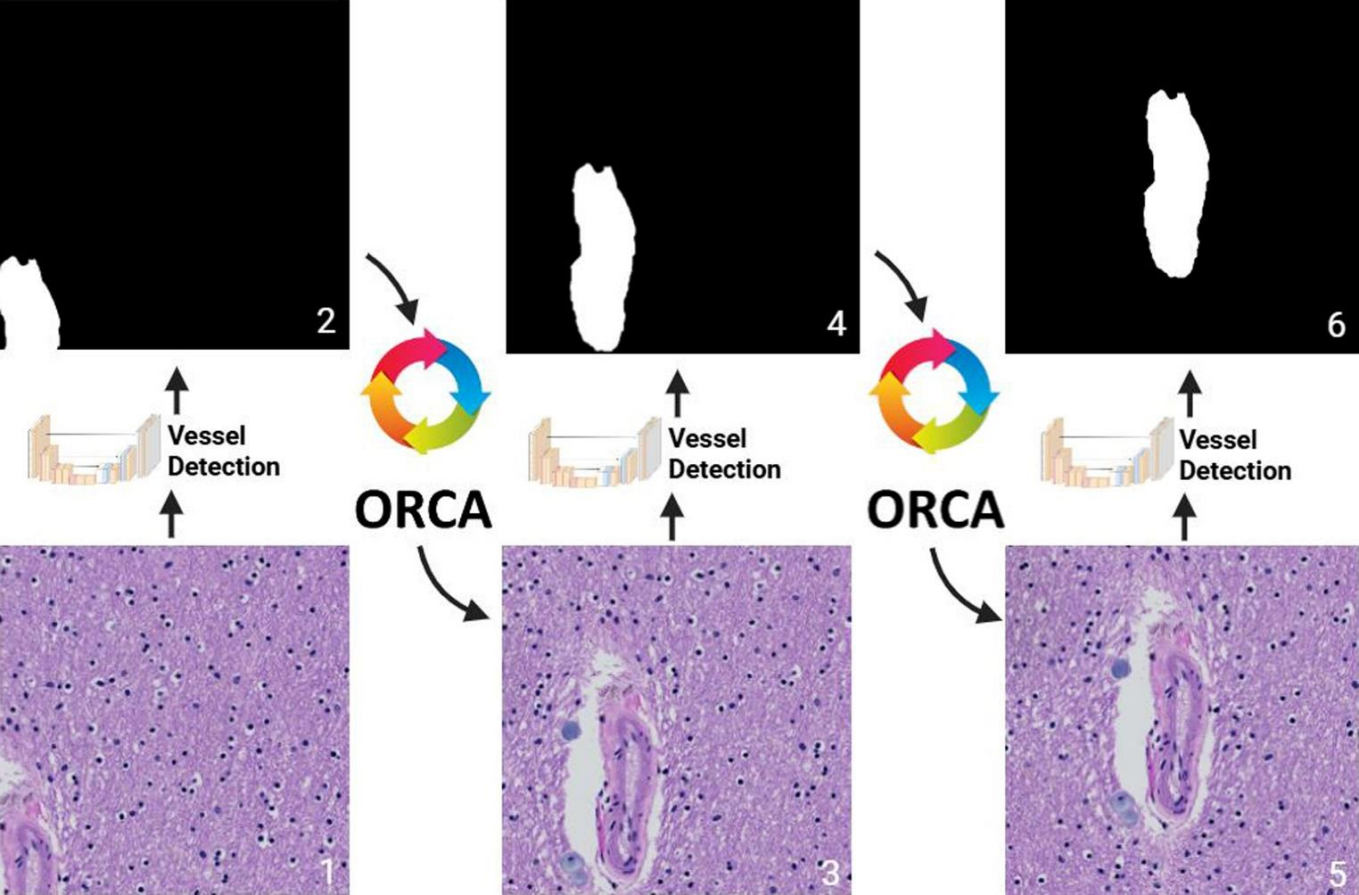
Starting at step (**1**), the algorithm inputs a raw (512 x 512) image tile through the embedded blood vessel detection model, which produces an output segmentation (**2**). (**3**) ORCA creates a new patch from the input WSI using modified shifted coordinates based on the previous segmentation. (**4–6**) Steps 1 through 3 are repeated until the vessel is centered.

Blood vessel centering was achieved by a custom recursive algorithm – Object of interest Recursive Centering Algorithm (ORCA) – wrapping the blood vessel detection neural network (**Figure 3**). The wrapper algorithm inputs raw (512 x 512) tiles into the blood vessel detection neural network, which segments blood vessels. Subsequently, the wrapper algorithm generates a new (512 x 512) tile with shifted boundaries such that the detected blood vessel resides closer to the center of the tile. This process is repeated until the detected blood vessel lies in the center of the final output tile (**Figure 3**). ORCA detects when the patch has been centered onto the blood vessel(s) by comparing the coordinates of the new patch with shifted boundaries to the original input patch; if the shift in boundaries is less than a preset threshold, then the patch is considered blood vessel(s) centered. The average runtime per WSI was approximately 37 minutes (see hardware section for further details).

#### Phase 1c: Arteriolosclerosis classification

 The arteriolosclerosis classification neural network consisted of an EfficientNetV2L^33^ backbone with five semi-trainable convolution layers topped by two fully trainable convolution layers, followed by three dense layers. 

#### Phase 1d: Arteriolosclerotic vessel segmentation

The arteriolosclerotic vessel segmentation network used the same architecture as the model for blood vessel detection.

#### Implementation of ML models

See **Supplementary Figure 3** for detailed architecture and **Supplementary Document 3** for detailed training hyperparameters of ML models in Phases 1a, 1b, 1c, and 1d.

#### Phase 2: Vascular Morphometry (VasMorph)

 For each blood vessel analyzed, VasMorph outputs the sclerotic index, vessel wall thickness, and vessel wall to lumen area ratio, which have previously been used as an indicator of the degree of vascular stenosis.^17^ The final sclerotic index and vessel wall thickness outputs include the median, mean, standard deviation, minimum, and maximum of sclerotic indices and vessel wall thicknesses calculated in a 360-degree rotation around the center of the blood vessel lumen. 

#### Sclerotic index

**Figure 4: Sclerotic index calculation:**
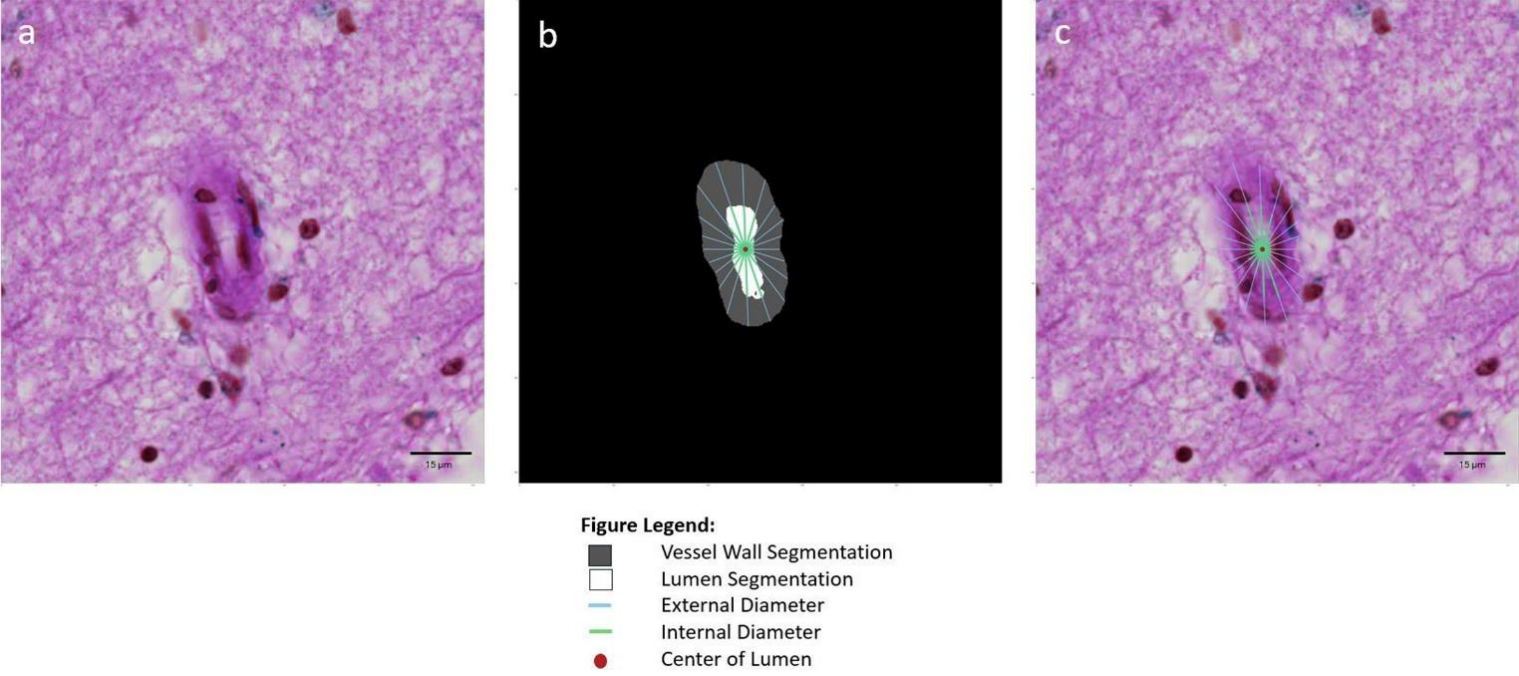
An image patch (**a**) centered onto an arteriolosclerotic blood vessel is input into the segmentation model of ArtSeg, which outputs the vessel wall and lumen segmentation (**b**). VasMorph measures the internal diameter (*D_i(θ)_*) and external diameter (*D_e(θ)_*) of the vessel to calculate the sclerotic index (*SI_θ_*) at each degree angle over a 180-degree rotation in a half circle around the centroid^34^ of the lumen segmentation to obtain a set of sclerotic indices (**b** and **c**). The output of VasMorph is the median, mean, standard deviation, minimum, and maximum of this set of vessel wall thicknesses. VasMorph finds the center of the lumen segmentation.

 To calculate the vessel sclerotic index, we first defined the sclerotic index at an angle theta (*SI_θ_*), given the internal diameter (*D_i(θ)_*) and external diameter (*D_e(θ)_*) of the vessel at that angle, using the following formula: *SI_θ_* = 1 – (*D_i(θ)_ / D_e(θ)_*).^11,12,24^ VasMorph then measures the internal diameter (*D_i(θ)_*) and external diameter (*D_e(θ)_*) of the vessel to calculate the sclerotic index (*SI_θ_*) at each degree angle over a 180-degree rotation in a half circle around the centroid^34^ of the lumen segmentation to obtain a set of sclerotic indices (**Figure 4**). The output of VasMorph is the median, mean, standard deviation, minimum, and maximum of this set of vessel sclerotic indices. 

#### Vessel wall thickness: Tangent line-based method

**Figure 5: Vessel wall thickness based on a line tangent to every point on the lumen contour:**
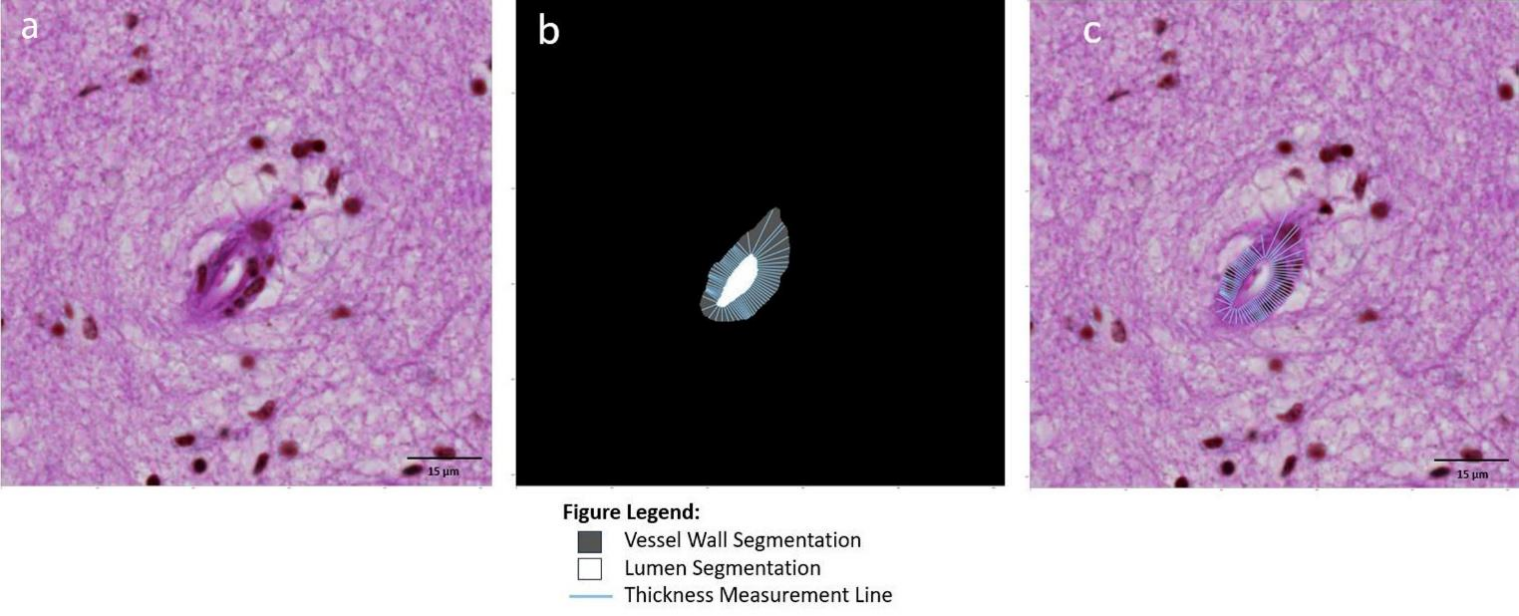
An image patch (**a**) centered onto an arteriolosclerotic blood vessel is input into the segmentation model of ArtSeg, which outputs the vessel wall and lumen segmentation (**b**). VasMorph measures the vessel wall thickness at each point in the lumen contour to obtain a set of thicknesses, where thickness at point alpha (*T_α_*) is defined as the distance between a line tangent to point alpha (*L_α_*) on the curve of the lumen contour and the external contour of the vessel (*E*): *T_α_* = E – *L_α_* (**b** and **c**). VasMorph then outputs the median, mean, standard deviation, minimum, and maximum of this set of vessel wall thicknesses.

 To calculate the vessel wall thickness, we first defined vessel wall thickness at point alpha (*T_α_*) on the curve lumen contour as the distance between a line tangent to point alpha (*L_α_*) and the external contour of the vessel (*E*): *T_α_* = E – *L_α_* (**Figure 5**). VasMorph then measures the vessel wall thickness at 100 equally spaced points in the lumen contour to obtain a set of thicknesses (**Figure 5**). The output of VasMorph is the median, mean, standard deviation, minimum, and maximum of this set of vessel wall thicknesses. 

#### Vessel wall thickness: Radii-based method

**Figure 6: Vessel wall thickness based on difference between internal and external radii:**
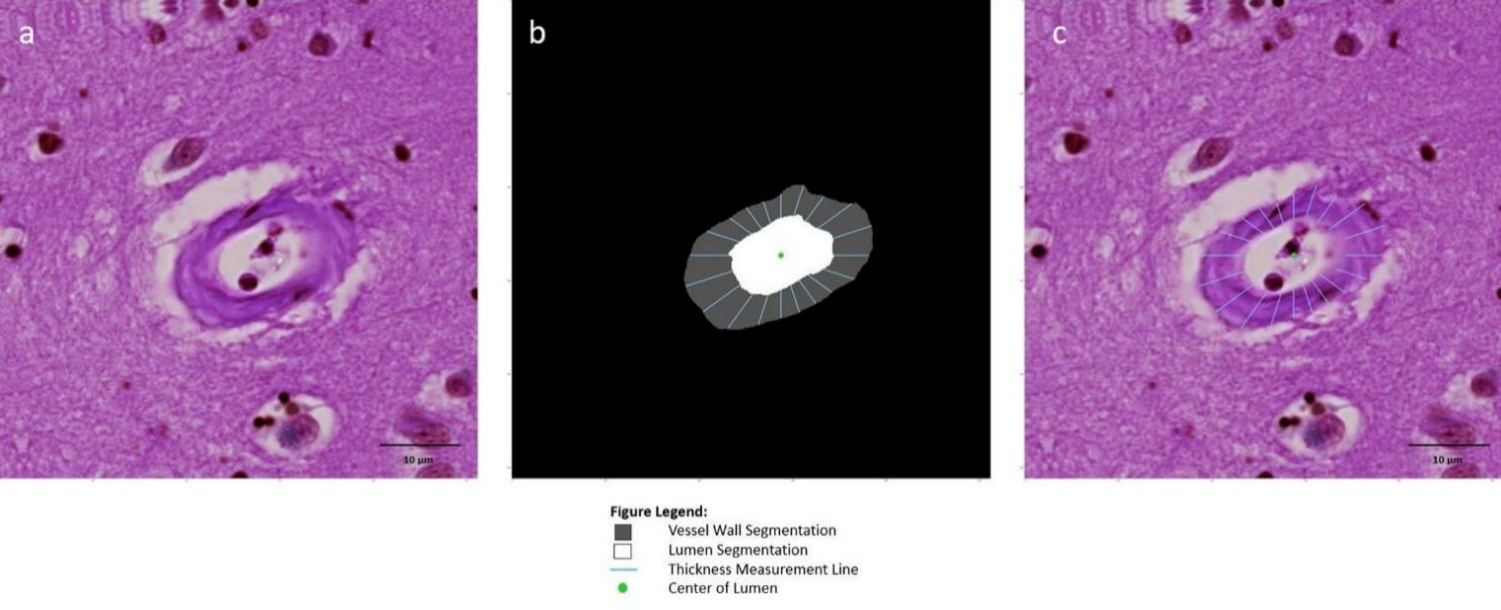
An image patch (**a**) centered onto an arteriolosclerotic blood vessel is input into the segmentation model of ArtSeg, which outputs the vessel wall and lumen segmentation (**b**). VasMorph measures the internal radius (*R_i(θ)_*) and external radius (*R_e(θ)_*) of the vessel to calculate the sclerotic index (*SI_θ_*) at each degree angle over a full 360-degree rotation around the centroid^34^ of the lumen segmentation to obtain a set of thicknesses, where thickness at angle theta (*T_θ_*) relative to the lumen center is defined as the difference between the external radius at theta (*R_e(θ)_*) and the internal radius at theta (*R_i(θ)_*): *T_θ_* = *R_e(θ) – _R_i(θ)_* (**b** and **c**). VasMorph then outputs the median, mean, standard deviation, minimum, and maximum of this set of vessel wall thicknesses.

 We also considered an alternative definition of vessel wall thickness at angle theta (*T_θ_*) relative to the lumen center as the difference between the external radius at theta (*R_e(θ)_*) and the internal radius at theta (*R_i(θ)_*): *T_θ_* = *R_e(θ) – _R_i(θ)_* (**Figure 6**). VasMorph then measures the internal radius (*R_i(θ)_*) and external radius (*R_e(θ)_*) of the vessel to calculate the sclerotic index (*SI_θ_*) at each degree angle over a full 360-degree rotation around the centroid^34^ of the lumen segmentation to obtain a set of thicknesses (**Figure 6**). VasMorph outputs the median, mean, standard deviation, minimum, and maximum of this set of vessel wall thicknesses based on this alternative definition. 

#### Vessel wall area to lumen area ratio

We calculated the vessel wall area to lumen area ratio by counting the number of pixels in the vessel wall segmentation and the lumen segmentation, then converting from pixels to μm^2^ using the conversion factor 0.0484 μm^2^ / pixel, where the length and height of each pixel is 0.220 μm, and finally dividing to obtain the ratio. By coincidence, the 62 vessels that fulfilled inclusion criteria were derived from UCD WSIs, which were scanned at 0.220 μm per pixel. The setting for the length and height of each pixel may be adjusted to match the WSI resolution.

#### Hardware

All scripts and python 3 modules for this project were run on the compute environment provided by the Center for Artificial Intelligence in Diagnostic Medicine at UCI which includes a high-end cluster of 88 NVIDIA GPU hardware accelerators composed of A100 (80 GB x16), A40 (40 GB x8), RTX Titan (24 GB x12), GTX Titan (16 GB x4), and GeForce RTX 2080 Ti (11 GB x48) graphics cards. WSIs and other data components were stored on a total of four CPU-optimized cluster nodes and three dedicated 0.24 PB file servers that are all interconnected on a high-speed 25 Gbps local fiber optic network.

## Results

 Our internal hold-out test set contains WSIs from the same institutions (UCI and UCD) and annotators (JJL, HPW, and KN) as WSIs used for model training and test internal deployment of ArtSeg. The external test set contains WSIs from a different institution (UCLA) and annnotator (VP) from WSIs used for model training and tests external deployment of ArtSeg. Hausdorff distance is converted from pixels to μm using a conversion factor of 0.220 μm / pixel (**Supplementary Table 1**). 

### Phase 1a: Detection of blood vessels on WSIs (ArtSeg)

 The initial blood vessel detection model serves as a preprocessing step, providing coarse segmentation to localize and center patches containing blood vessels. The marginally lower Dice score observed is consistent with the annotation quality at this stage and is adequate for the intended purpose of blood vessel detection. Fine segmentation is achieved at a later stage (**Phase 1d**) during the segmentation of arteriolosclerotic blood vessel walls and lumens. 

#### Internal hold-out testing

**Figure 7: ROC curves of hold-out and external tests for the vessel detection:**
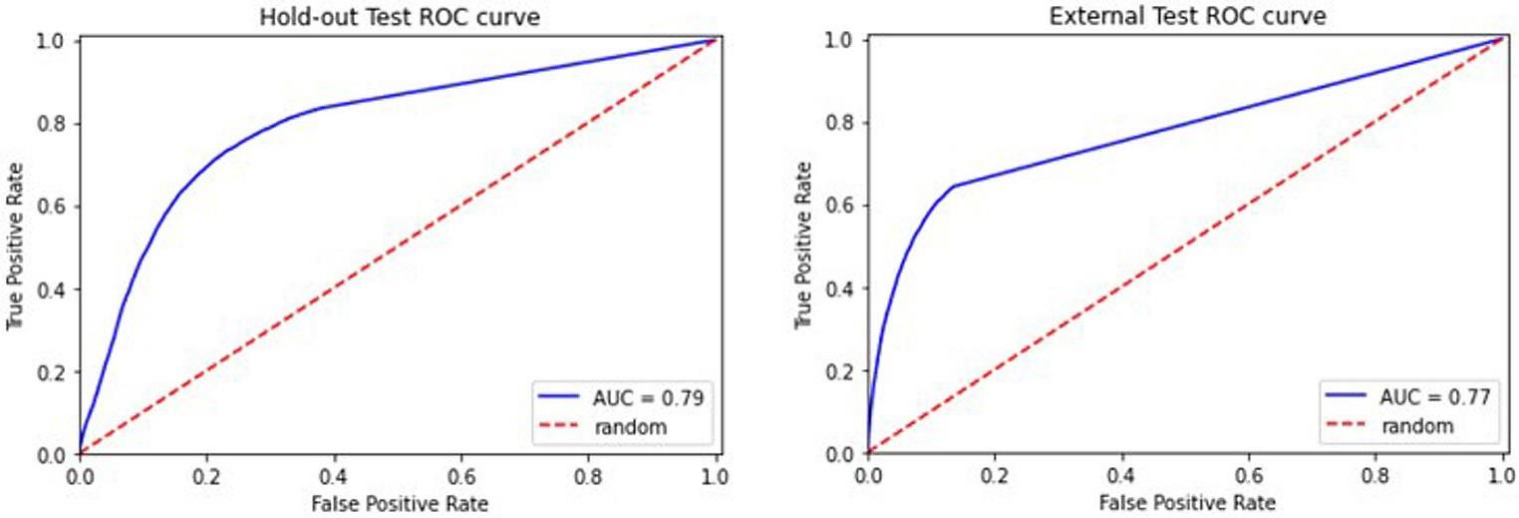
As expected, the model performs slightly worse for the external test. Vessel detection model acts as a screening tool to quickly remove patches without vessels while retaining patches with vessels or objects suspicious of vessels.

 After converting the segmentation output of the model into classification confidence scores, the area under the receiver operating characteristic curve (AUC-ROC) of the model is 0.79 (**Figure 7**). The vessel detection segmentation model achieved a dice score of 0.56 and a Hausdorff distance of 0.56 μm on the hold-out test (**Table 1**). 

#### External stress testing

 After converting the segmentation output of the model (obtained by running inferences on an external cohort) into classification confidence scores, the area under the receiver operating characteristic curve (AUC-ROC) of the model is 0.77 (**Figure 7**). The vessel detection segmentation model achieved a dice score of 0.74 and a Hausdorff distance of 0.47 μm on the external test (**Table 1**). 

**Table 1:** Results of vessel detection segmentation internal hold-out and external testing

*Dataset*	Dice score	Hausdorff distance (μm)
*Hold-out*	0.56	0.56
*External Test*	0.74	0.47

### Phase 1b: Object of interest centering through recursive segmentation and boundary migration (ORCA)

**Figure 8:**
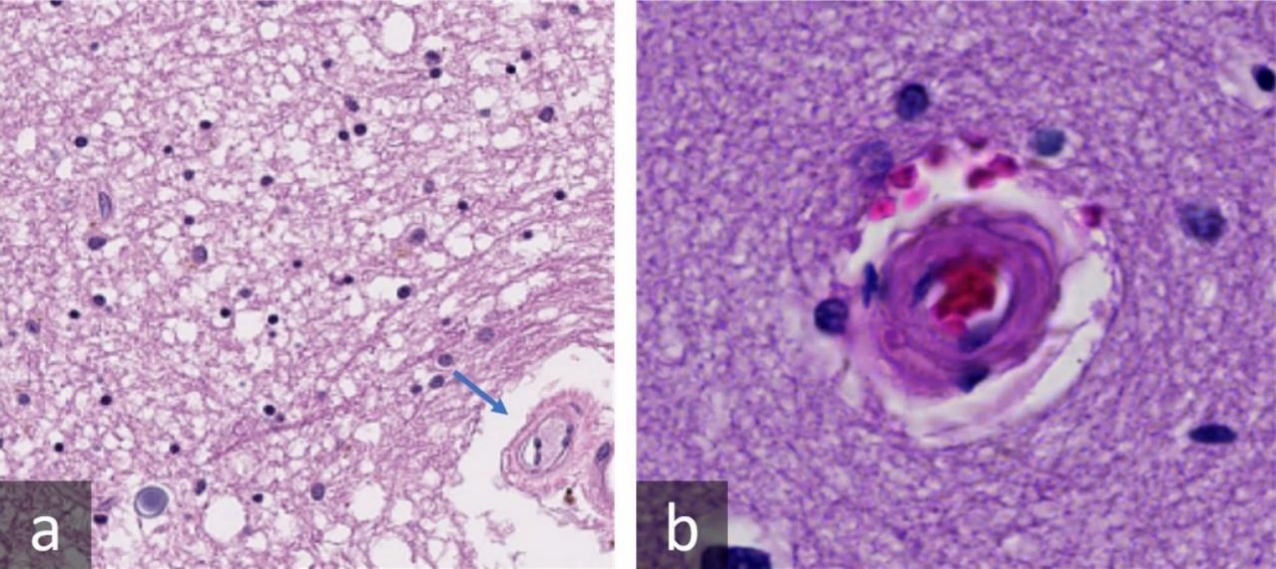
**Figure 8: Results of vessel centering by ORCA.** (**a**) WSI tiling generates tiles that often contain blood vessel(s) at the tile edge, with some vessel cropped (arrow). (**b**) ORCA generates new tiles with the detected blood vessel(s) located in the center.

 Segmentation CNNs such as U-Net operate most effectively when the object of interest (OI) is fully visible and not cropped. The process of WSI tiling occurs irrespective of the positions of OIs, frequently creating cropped OIs that appear on the edge of the tile (**Figure 8**). We hypothesized that cropping of blood vessels reduced segmentation performance and that centering tiles onto blood vessels would improve performance. 

 To achieve OI centering, we designed a custom recursive algorithm – Object of Interest Recursive Centering Algorithm (ORCA) – wrapping our blood vessel identification neural network. From 16 WSIs, ORCA generated 401 tiles with arteriolosclerotic blood vessels and 7066 tiles with non-arteriolosclerotic blood vessels or without vessels. Arteriolosclerosis was defined using criteria proposed by Skrobot et al.^8,10^ Visualization of tiles demonstrated centering onto blood vessels (**Figure 8**). These vessel-centered tiles were utilized to train, validate, and test the arteriolosclerosis classification and arteriolosclerotic vessel segmentation ML models. 

### Phase 1c: Arteriolosclerosis classification is feasible, albeit challenging (ArtSeg)

#### 3-fold cross validation

 For the classification of arteriolosclerosis, our model achieved a mean validation set performance of 0.94 accuracy and 0.68 AUC-ROC (**Table 2**). 

**Table 2:** Results of arteriolosclerosis classification 3-fold cross-validation

*K*	Specificity	Sensitivity (recall)	Precision	Accuracy	F1	AUC-ROC
*0*	1.00	0.19	0.68	0.96	0.29	0.59
*1*	0.95	0.52	0.23	0.94	0.32	0.73
*2*	0.97	0.51	0.67	0.91	0.58	0.74
*Mean*	0.97	0.37	0.47	0.94	0.38	0.68

#### Internal hold-out testing

 Our internal hold-out test set, which was not seen by the model during training, consisted of 4 cases from 2 participants, 1 each from UCD and UCI, respectively, labeled by novice annotators JJL, HPW, and KN, who also labeled the training data set. For the classification of arteriolosclerosis, our model achieved an accuracy of 0.86 and an AUC-ROC of 0.87 (**Table 3**). 

#### External stress testing

 Our external stress test set, which was not seen by the model during training, consisted of 2 cases from 2 participants, both from UCLA, labeled by expert annotator VP, who did not label the training data set. For the classification of arteriolosclerosis, our model achieved an accuracy of 0.77 and an AUC-ROC of 0.83 (**Table 3**). 

**Table 3:** Results of arteriolosclerosis classification internal hold-out and external testing

*K*	Specificity	Sensitivity (recall)	Precision	Accuracy	F1	AUC-ROC
*Hold-out*	0.856	0.89	0.13	0.86	0.23	0.87
*External Test*	0.752	0.92	0.26	0.77	0.41	0.83

### Phase 1d: Attention U-Net effectively segments the arteriolosclerotic vessel walls and lumen (ArtSeg)

#### 3-fold cross validation

 For the segmentation of vessel *walls and lumens* (**Figures 9–12**), our model achieved a mean validation set performance of 0.68 Dice score, 1.68 μm Hausdorff distance, and 0.90 AUC-ROC (**Table 4**). For the segmentation of vessel *walls only*, our model achieved validation set performance of 0.69 Dice score, 1.41 μm Hausdorff distance, and 0.86 AUC-ROC (**Table 5**). For the segmentation of the vessel *lumen only*, our model achieved validation set performance of 0.63 Dice score, 0.99 μm Hausdorff distance, and 0.86 AUC-ROC (**Table 6**). 

**Table 4:** Results of arteriolosclerotic vessel segmentation 3-fold cross-validation for vessel *walls and lumens*

*Dataset*	Dice score	Hausdorff distance (μm)
*0*	0.72	1.56
*1*	0.70	1.63
*2*	0.63	1.87
*Mean*	0.68	1.68

**Table 5:** Results of arteriolosclerotic vessel segmentation 3-fold cross-validation for vessel *walls only*

*Dataset*	Dice score	Hausdorff distance (μm)
*0*	0.71	1.36
*1*	0.71	1.34
*2*	0.65	1.54
*Mean*	0.69	1.41

**Table 6:** Results of arteriolosclerotic vessel segmentation 3-fold cross-validation for vessel *lumen only*

*Dataset*	Dice score	Hausdorff distance (μm)
*0*	0.72	0.97
*1*	0.63	0.95
*2*	0.54	1.06
*Mean*	0.63	0.99

**Figure 9: Example segmentation results for blood vessels with mild arteriolosclerosis as classified by a neuropathology fellow (JL) and annotated by non-experts (KN, HSW, JL).**
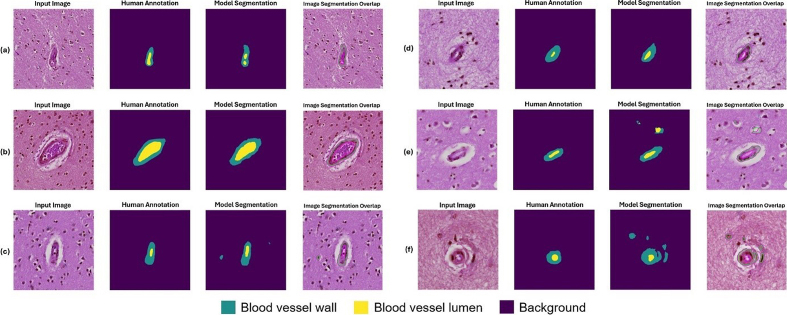
Six example instances with input image, human annotation mask, model segmentation output, and an overlap image of input image and model segmentation are shown here. The blood vessels shown here were classified as having arteriolosclerosis by a human annotator. (**a**, **b**) Example instances of good model performance. (**c**, **d**) Example instances of intermediate model performance. (**e**, **f**) Example instances of poor model performance.

**Figure 10: Example segmentation results for blood vessels with moderate arteriolosclerosis as classified by a neuropathology fellow (JL) and annotated by non-experts (KN, HSW, JL).**
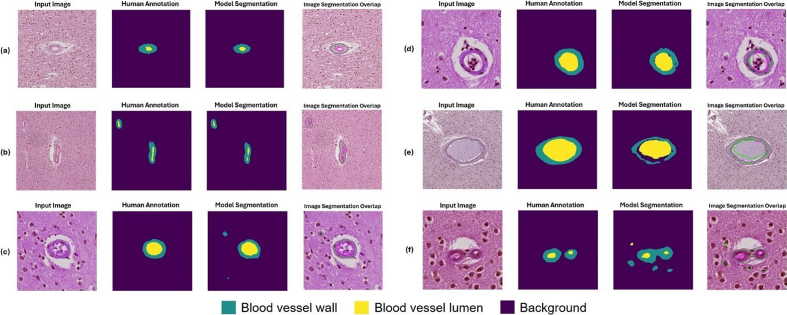
Six example instances with input image, human annotation mask, model segmentation output, and an overlap image of input image and model segmentation are shown here. The blood vessels shown here were classified as having arteriolosclerosis by a human annotator. (**a**, **b**) Example instances of good model performance. (**c**, **d**) Example instances of intermediate model performance. (**e**, **f**) Example instances of poor model performance.

**Figure 11: Example segmentation results for blood vessels with severe arteriolosclerosis as classified by a neuropathology fellow (JL) and annotated by non-experts (KN, HSW, JL).**
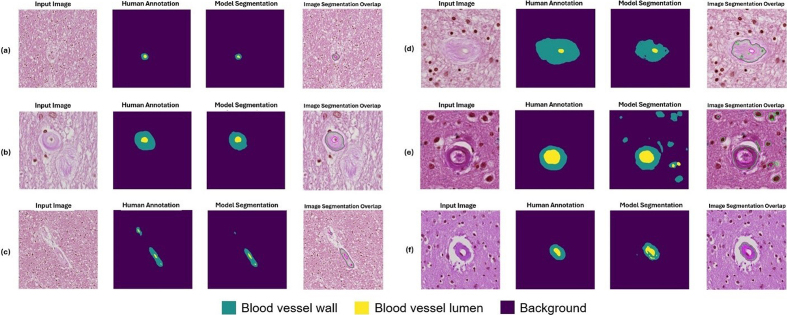
Six example instances with input image, human annotation mask, model segmentation output, and an overlap image of input image and model segmentation are shown here. The blood vessels shown here were classified as having arteriolosclerosis by a human annotator. (**a**, **b**) Example instances of good model performance. (**c**, **d**) Example instances of intermediate model performance. (**e**, **f**) Example instances of poor model performance.

**Figure 12: Example segmentation results for challenging instances as classified by a neuropathology fellow (JL) and annotated by non-experts (KN, HSW, JL).**
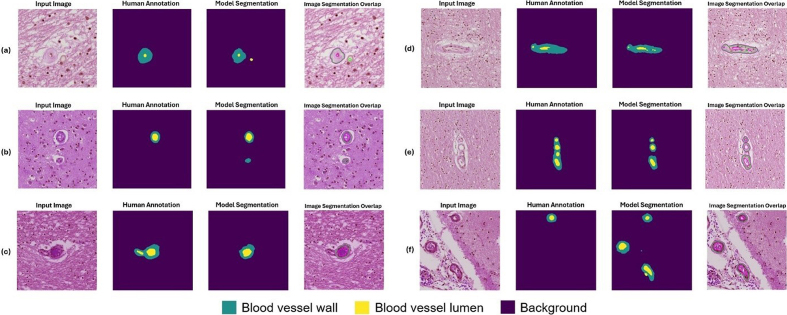
Six example instances with input image, human annotation mask, model segmentation output, and an overlap image of input image and model segmentation are shown here. The blood vessels shown here were classified as having arteriolosclerosis by a human annotator. (**a**) A corpora amylacea was mistaken for a vessel lumen by ArtSeg. (**b**) ArtSeg misidentified a non-arteriolosclerotic vessel as having arteriolosclerosis and a target for segmentation. (**c**) Conversely, a vessel with arteriolosclerosis is misidentified as a non-arteriolosclerotic vessel and omitted for segmentation. (**d**) ArtSeg fails to identify moderate hyaline vessel wall thickening and lumen stenosis. (**e**) Image tiles with clusters of blood vessels present a particular challenge to ArtSeg and VasMorph. (**f**) Vessels within the leptomeninges are mistakenly segmented by ArtSeg.

#### Internal hold-out testing

 Our internal hold-out test set, which was not seen by the model during training, consisted of 4 cases from 2 participants, 1 each from UCD and UCI, respectively, labeled by novice annotators JJL, HPW, and KN, who also labeled the training data set. For the segmentation of vessel *walls and lumen*, our model achieved a 0.73 Dice score, 1.52 μm Hausdorff distance, and 0.92 AUC-ROC (**Table 7**). For the segmentation of vessel *walls only*, our model achieved a 0.73 Dice score, 1.30 μm Hausdorff distance, and 0.88 AUC-ROC (**Table 8**). For the segmentation of the vessel *lumen only*, our model achieved a 0.71 Dice score, 0.88 μm Hausdorff distance, and 0.90 AUC-ROC (**Table 9**). 

#### External stress testing

 Our external stress test set, which was not seen by the model during training, consisted of 2 cases from 2 participants, both from UCLA, labeled by expert annotator VP, who did not label the training data set. For the segmentation of vessel *walls and lumen*, our model achieved a 0.71 Dice score, 1.72 μm Hausdorff distance, and 0.87 AUC-ROC (**Table 7).** For the segmentation of vessel *walls only*, our model achieved a 0.70 Dice score, 1.36 Hausdorff distance, and 0.83 AUC-ROC (**Table 8**). For the segmentation of the vessel *lumen only*, our model achieved a 0.65 Dice score, 1.03 μm Hausdorff distance, and 0.80 AUC-ROC (**Table 9**). 

**Table 7:** Results of arteriolosclerotic vessel segmentation internal hold-out and external testing for vessel *walls and lumens*

*Dataset*	Dice score	Hausdorff distance (μm)
*Hold-out*	0.73	1.52
*External Test*	0.71	1.72

**Table 8:** Results of arteriolosclerotic vessel segmentation internal hold-out and external testing for vessel *walls only*

*Dataset*	Dice score	Hausdorff distance (μm)
*Hold-out*	0.73	1.30
*External Test*	0.70	1.36

**Table 9:** Results of arteriolosclerotic vessel segmentation internal hold-out and external testing for vessels' *lumens only*

*Dataset*	Dice score	Hausdorff distance (μm)
*Hold-out*	0.71	0.88
*External Test*	0.65	1.03

### Phase 2: Vascular Morphometry (VasMorph)

#### Sclerotic index calculation

 The mean and median of the sclerotic indices of 62 vessels in the test set calculated by VasMorph were 0.53 and 0.53, respectively. The standard deviation of the sclerotic indices was 0.05. The minimum was 0.43 and the maximum was 0.60. 

#### Vessel wall thickness

 The vessel wall thickness metrics (mean, median, standard deviation, minimum, and maximum) for the manual measurements, tangent line-based method, and radii-based method are outlined in **Table 10**. In this preliminary comparison of the two methods, the tangent line-based method appears to generate metrics with greater proximity to manual measurements. 

**Table 10:** Comparison of the tangent line-based method and the radii-based method to manual measurements

*Vessel Wall Thickness Metrics*	Manual Measurement	Tangent Line-Based Method	Radii-Based Method
*Mean*	24.71	23.97	52.59
*Median*	23.13	22.57	49.16
*Standard Deviation*	8.37	6.89	12.97
*Minimum*	17.00	14.41	36.74
*Maximum*	35.60	45.38	80.31

#### Vessel wall to lumen area ratio

 The mean vessel wall area, lumen area, and vessel wall to lumen area ratio of 62 vessels in the test set, calculated by VasMorph, were 84.79 μm^2^, 286.8 μm^2^, 3.38 μm^2^, respectively. 

## Discussion

 In this study, we present a novel proof of concept ML pipeline (ArtSeg) capable of automatically detecting blood vessels, classifying blood vessels by presence or absence of arteriolosclerosis, and segmenting arteriolosclerotic blood vessel walls and lumens. Furthermore, we introduce a wrapper algorithm (ORCA) that centers tiles onto objects of interest; and a custom algorithm (VasMorph) that calculates the sclerotic index, vessel wall thickness, and vessel wall to lumen area ratio from arteriolosclerotic blood vessel segmentations. 

 ArtSeg performs four sequential steps: (1) blood vessel detection, (2) blood vessel centering, (3) arteriolosclerosis classification, and (4) arteriolosclerotic vessel segmentation (see **Figure 2** for overview). First, we trained an Attention Unet-based neural network to segment and identify blood vessels(s) within patches produced by WSI tiling. Second, patches containing non-centered blood vessel(s) are then fed into ORCA to produce new patches centered onto the detected blood vessel(s). Third, a binary classification model was trained to separate patches with arteriolosclerotic blood vessel(s) from those without. Fourth, we trained an ML model to segment walls and lumens of arteriolosclerotic blood vessels. The segmentation outputs of ArtSeg are input into VasMorph to obtain the sclerotic index, vessel wall thickness, and vessel wall to lumen area ratio of each arteriolosclerotic blood vessel. 

 As a component of ArtSeg, we developed a novel object of interest recursive centering algorithm (ORCA), which may be applied to any object of interest and not just blood vessels. ORCA wraps a segmentation model, which may be customized to fit the target task. ORCA first inputs raw image tiles generated by WSI tiling into the segmentation model, which segments the object of interest. Based on this segmentation output, ORCA generates a new image patch with boundaries shifted such that the detected object of interest resides closer to the center of the tile. This process is repeated until the detected object of interest lies in the center of the final output tile (**Figure 3**). 

 When building VasMorph, we encountered a dearth of literature on the mathematical definition of blood vessel wall thickness. We utilized the following definition: the distance between the inner boundary of the vessel endothelium and the outer boundary of the tunica adventitia along a line perpendicular to the wall’s “backbone” or minimum skeleton – the arc equidistant from the outer and inner wall boundaries, which equates to the sum of the widths of the endothelium, tunica intima, tunica media, and tunica adventitia.^35^ Because we cannot calculate the equation for the minimum skeleton, and local fluctuations make estimates of its slope unreliable, indirect methods to approximate the wall thickness have been described.^35^ While using the difference between the outer vessel radius and the inner vessel radius has been described, our method of finding the line perpendicular to a line tangent to each point along the lumen contour (tangent line-based method) has not been previously proposed to the best of our knowledge. In our preliminary study, we find that our tangent line-based method produces metrics comparable to expert annotations. 

**Figure 13: Involutions in the lumen contour produce inaccurate thickness measurement lines when using the tangent line-based method.**
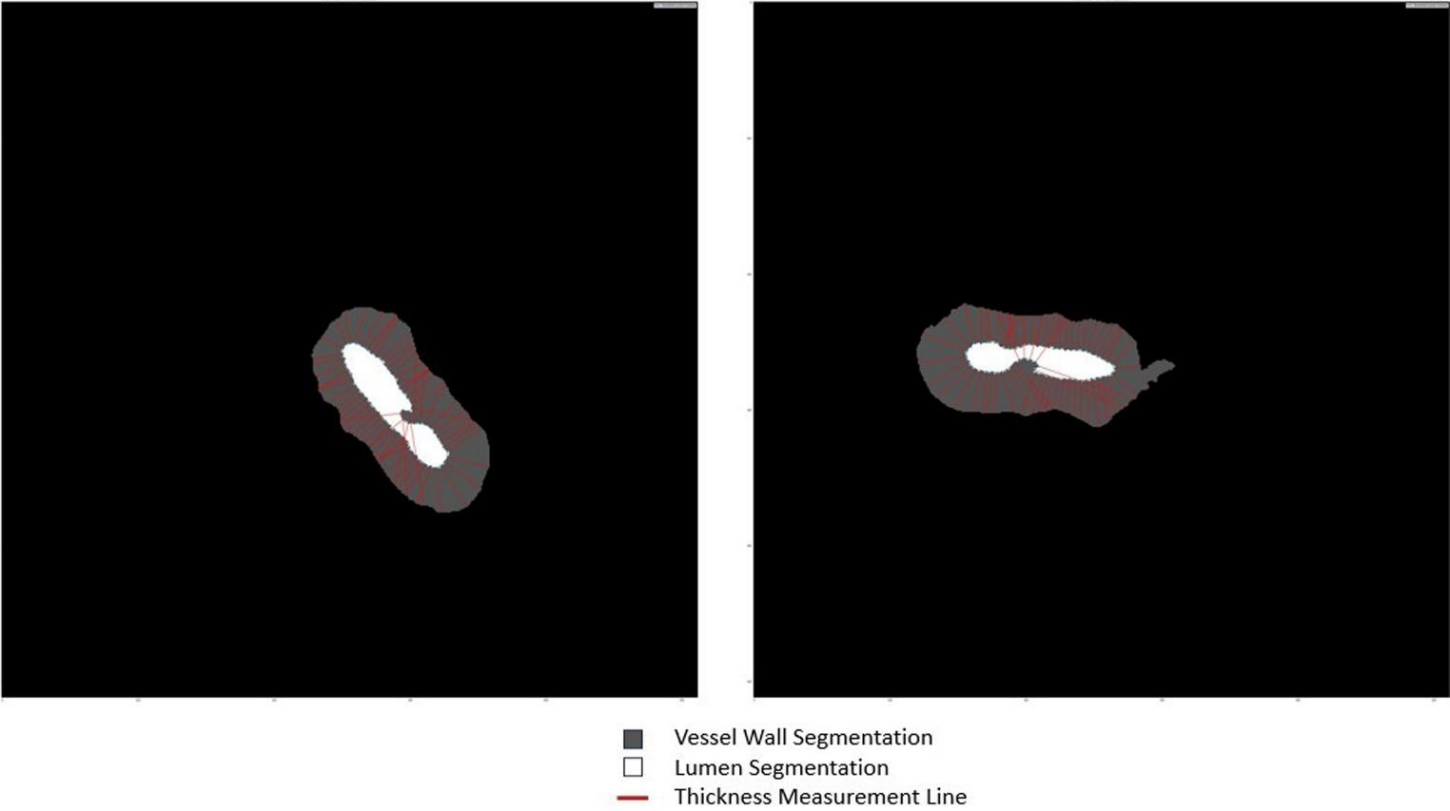
Because the thickness measurement line is extrapolated from the lumen contour, these involutions cause the thickness measurement line to point towards the lumen center and to traverse across it.

**Figure 14: Images with multiple contiguous vessels pose a particular challenge to the current version of VasMorph.**
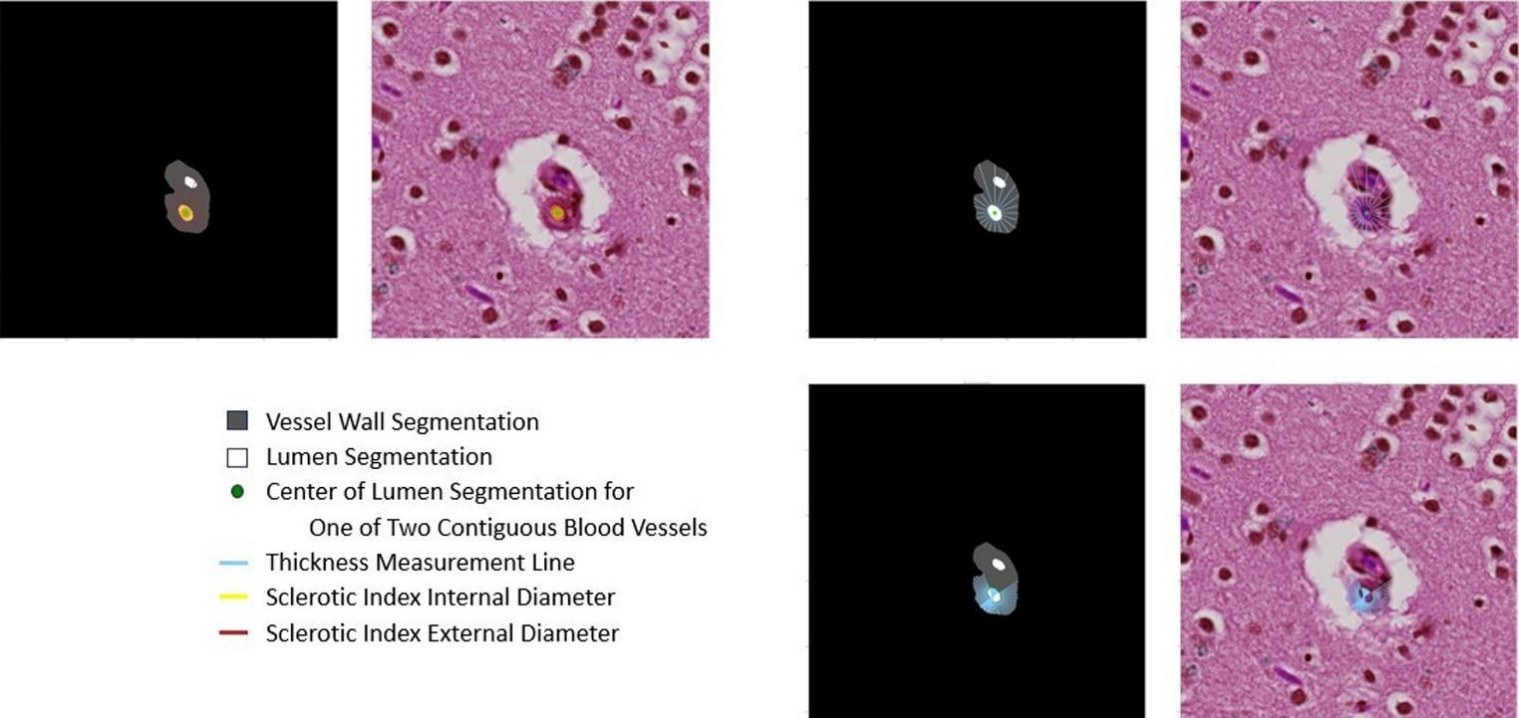
ArtSeg is unable to discern the border between the two blood vessels. The sclerotic index calculation depends upon having the complete circumference of the blood vessel's external contour to serve as an outer bound for the external diameter.

 There are several notable areas for improvement in VasMorph. First, the tangent line-based method for calculating vessel wall thickness mishandles lumens with highly convoluted and irregular contours (**Figure 13**). Second, the current version of VasMorph can only accept image tiles containing only one vessel. Image tiles with more than one vessel cannot yet be analyzed by VasMorph (**Figure 14**) because such image tiles often contain multiple vessels with contiguous vessel walls. Finally, VasMorph currently does not correct for eccentricity of blood vessels due to the angle of sectioning through the vessel. 

 The method to calculate vessel wall thickness using a tangent line tends to mishandle irregular lumens. Rare blood vessels with lumens that contain an involution in the lumen contour (**Figure 13**) pose a significant challenge to the current version of VasMorph. Because the thickness measurement line is extrapolated from the lumen contour, these involutions cause the thickness measurement line to point towards the lumen center and to traverse across it (**Figure 13**). The simplest solution for future implementations of VasMorph would be to exclude any thickness measurement line that points towards the center of the lumen. 

 VasMorph cannot process multi-vessel images because the segmentation output of ArtSeg does not currently distinguish between two blood vessels with contiguous vessel walls (**Figure 14**). ArtSeg is unable to discern the border between the two blood vessels. The sclerotic index calculation depends upon having the complete circumference of the blood vessel's external contour to serve as an outer bound for the external diameter. Similarly, both methods for calculating vessel wall thickness also require the complete circumference of the blood vessel external contour to serve as the outer bound for the external radius (radii-based method) and the thickness measurement line (tangent line-based method). Several solutions are possible: (1) teach ArtSeg to differentiate between two vessels with contiguous vessel walls by labeling each vessel as a separate object, or (2) exclude measurements that require external contour at the region of vessel wall contiguity. 

 VasMorph may be improved by adding a correction for eccentric sectioning of blood vessels. A section through a cylindrical vessel at angle θ will result in an ellipsis with a short (*d_s_*) and long (*d_l_*) diameter. The ratio of *d_s_* and *d_l_* equals the cosine of θ, which can be used as a correction factor. The current version of VasMorph presented in this paper has yet to implement this type of correction. 

 To the best of our knowledge, ArtSeg is the first open-source ML-based pipeline for the classification of arteriolosclerosis in blood vessels and the segmentation of arteriolosclerotic blood vessel walls and lumens in WSIs of the brain. ORCA is the first ML-based algorithm capable of generating image patches centered on objects of interest in human post-mortem brain tissue. And VasMorph is the first algorithm for automated calculation of sclerotic index, vessel wall thickness, and vessel wall to lumen area ratio using blood vessel segmentations. All three frameworks represent scalable prototype methodologies to achieve their respective goals and serve as blueprints for further refinement and improvement. Notably, ArtSeg is model-agnostic, meaning ML models contained within the algorithm may be replaced and updated as novel state-of-the-art architectures are discovered. 

 Several caveats merit mention. First is the small sample size of only 13 participants included in this proof-of-concept study. Blood vessel morphology may vary from individual to individual, and scaling up ArtSeg will likely require further training with WSIs from hundreds to thousands of disparate participants. Although small, the external validation cohort offers preliminary support for the algorithm’s performance. These promising pilot results warrant further validation with larger cohorts and additional WSIs. We do want to acknowledge obtaining high-quality annotated datasets is labor-intensive and time-consuming. Second, only three brain banks (UC Davis, UCI, and UCLA) within California were involved in this study, which may not represent the diversity of cerebrovascular pathology, staining methods, and slide digitization protocols seen in brain banks across the United States. More diverse datasets spanning multiple institutions will provide a more robust and generalizable ML-based algorithm. Third, all WSIs were derived from only two brain regions, the frontal lobe or the occipital lobe. We only examined blood vessels within the white matter. A broader sampling of brain regions will provide more representative datasets spanning the entire brain. Fourth, only 2 of the 13 participants in our cohort lacked arteriolosclerosis. The addition of more normal controls would strengthen the evaluation of the specificity and negative predictive value of the algorithm. Fifth, our preliminary testing of the two methods for vessel wall thickness calculation includes measurements made by a single neuropathologist; a robust test set would include multiple experts. Sixth, the current version of ArtSeg does not differentiate and is unable to discern the border between multiple contiguous blood vessels within the same image tile. This presents a significant limitation when calculating the sclerotic index, vessel wall thickness, and vessel wall to lumen area ratio, which require accurate contours of each blood vessel wall and lumen within the analyzed image tile. Seventh, we were only able to annotate each image tile once, and so we were unable to conduct a study on the inter-rater reliability of our annotations. Eighth, we did not explicitly examine the internal elastic lamina to differentiate arterioles from venules, although we applied criteria from Skrobot et al.^8,10^ to define arteriolosclerosis and referenced **Figure 3** in their paper as a guide for categorizing no, mild, moderate, and severe arteriolosclerosis. Our prototype algorithm analyzes only H&E-stained sections; the addition of protein-specific stains such as CD31 immunohistochemistry or Verhoeff–Van Gieson histochemistry may enhance visualization of specific vascular structures, including endothelial cells and the internal elastic lamina. And lastly, the ML models contained within the current version of ArtSeg have not had hyperparameters exhaustively optimized for peak performance. The prototype ArtSeg published here serves as a proof of concept to demonstrate the promising potential of our method, which provides a scalable blueprint for further refinement. Furthermore, the blood vessel detection model currently uses an EfficientNetV2L-based Attention U-net, which may be computationally expensive in comparison to YOLOv7,^36^ one of the most computationally efficient and accurate object detection models for computer vision tasks. 

 We specifically designed our training, internal hold-out, and external test sets to model real-world situations where the model may be deployed at an institution that did not contribute to its training dataset and evaluated by domain experts who did not participate in annotating the training dataset. The training and internal hold-out dataset consisted of WSIs from UCI and UCD labeled by annotators JJL, HPW, KN, whereas the external test set consisted of WSIs from UCLA annotated by attending neuropathologist VP, who did not see images in the training dataset. Our study demonstrates that ArtSeg performs slightly worse when applied to an external institution and evaluated by an external domain expert, but overall exhibits considerable resilience to these variables. 

 In the future, we anticipate collecting datasets from multiple sources annotated by multiple brain arteriolosclerosis experts to build a more robust and reliable ML pipeline. Another next step in algorithm validation would be to examine the correlation between the algorithm-derived sclerotic index and established semi-quantitative scales. Learning from our pilot experience, we will ask annotators to differentiate between multiple blood vessels within an image tile, especially contiguous vessels. We may validate our tangent line-based method of calculating vessel wall thickness through a more rigorous test set annotated by multiple brain arteriolosclerosis experts. ArtSeg may be further optimized by testing YOLOv7 for blood vessel detection as well as novel segmentation and classification architectures such as Segment Anything Model,^37^ SegFormer,^38^ EfficientViT,^39^ or CoCa.^40^ Another consideration is to expand ArtSeg to distinguish arteriolosclerosis from cerebral amyloid angiopathy on H&E-stained slides. To improve the ML-based tool, we plan to incorporate a quality assurance 'human-in-the-loop' step, allowing users to correct suboptimal segmentation outputs. We will assess its impact by comparing segmentation metrics before and after user supervision. During our labeling process, our annotators anecdotally observed subjectivity when applying the Skrobot et al. arteriolosclerosis criteria to vessels with mild arteriolosclerosis from those without, which warrants a dedicated follow-up study on the inter- or intra-rater variability of arteriolosclerosis classification. Future versions of ArtSeg and VasMorph may potentially mitigate this variability by introducing quantitative thresholds – such as sclerotic index, vessel wall thickness, and vessel wall to lumen area ratio – to define arteriolosclerosis. Finally, we plan to combine ArtSeg with another ML pipeline that screens for microinfarcts^41^ to create a comprehensive ML-based tool capable of analyzing the relationship between vascular morphology and microinfarcts.

## Conclusion

 Taken together, the present study demonstrates a pilot ML approach to assist persons in the morphometrical analysis of blood vessels in histopathological images. Our ML pipeline showed promising capabilities for inference on unseen WSIs from a disparate institution and annotator, which would be encountered in real-world deployment of such a tool. Within our pipeline, we present a generalizable novel algorithm capable of centering image tiles onto an object of interest. Furthermore, we propose a novel method to calculate vessel wall thickness and present preliminary data showing this method agrees with human interpretations of vessel wall thickness. Our pipeline is flexible as the ML models contained in the pipeline can be updated as novel state-of-the-art architectures are discovered by the artificial intelligence community. Furthermore, our approach provides preliminary evidence that breaking a complex task into multiple steps, each addressed by a separate machine learning model, may be the optimal path for the segmentation of pathological features of interest. We hope this proof of concept inspires further work in this field. We provide the code and dataset for our pipeline openly available to the community (see Data Availability and Code Availability). 

## Data availability

 All available data are located in Zenodo records listed within the GitHub repository (https://github.com/jerryjlou/ArtSeg-VasMorph). All WSIs used in this study are available in their raw, de-identified form. Preprocessing, training, and evaluation can be carried out using the codes listed in this manuscript.

## Code availability

 All code for ArtSeg and VasMorph can be found in the GitHub repository (https://github.com/jerryjlou/ArtSeg-VasMorph), which contains the full end-to-end pipeline. No outside code is necessary to reproduce the study results.

## Conflict of interest statement

The authors declare no competing interests.

## Supplementary Material

Supplementary Figures 1–3, Table 3–6 (PDF file, 1139 KB)

Supplementary Table 1 (xlsx file, 15 KB)

Supplementary Table 2 (xlsx file, 19 KB)

Supplementary Document 1 (docx file, 3510 KB)

Supplementary Document 2 (Sup Fig 4,5) (docx file, 428 KB)

Supplementary Document 3 (docx file, 108 KB)
