# Supplementary material for: Applying machine learning to assist in the morphometric assessment of brain arteriolosclerosis through automation
Source: Free Neuropathol. 2025 Jun 2;6:12. doi: 10.17879/freeneuropathology-2025-6387 (PMC12159543; doi:10.17879/freeneuropathology-2025-6387)
Supplement: Supplementary file 1 [file freeneuropathol-06-12-6387-s1.pdf]

Supplementary material

- **Supplementary Tables 3 – 6**
- **Supplementary Figures 1 – 3**

Supplementary Table 3

K	Number of tiles with arteriolosclerotic vessel(s)	Number of tiles without arteriolosclerotic vessel(s)
0	263, 108 (training, validation)	3905, 2314
1	294, 77	3607, 2612
2	185, 186	4926, 1293

Supplementary Tables 3: Summary of dataset for arteriolosclerosis classification 3-fold cross-validation

Supplementary Table 4

K	Number of tiles: training	Number of tiles: validation
0	263	108
1	294	77
2	185	186

Supplementary Tables 4: Summary of dataset for arteriolosclerotic vessel segmentation 3-fold cross-validation

Supplementary Table 5

Dataset	Number of Participants	Number of WSIs	Number of tiles with arteriolosclerotic vessel(s)	Number of tiles without arteriolosclerotic vessel(s)
Training	8	28	371	6210
Internal Hold-out	2	4	18	722
External Test	1	2	12	125

Supplementary Tables 5: Summary of dataset for arteriolosclerosis classification and segmentation hold-out and external testing

Copyright: © 2025 The author(s). This is an open access article distributed under the terms of the Creative Commons Attribution 4.0 International License (https://creativecommons.org/licenses/by/4.0/), which permits unrestricted use, distribution, and reproduction in any medium, provided the original author and source are credited, a link to the Creative Commons license is provided, and any changes are indicated. The Creative Commons Public Domain Dedication waiver (https://creativecommons.org/publicdomain/zero/1.0/) applies to the data made available in this article, unless otherwise stated.

Supplementary Table 6

Dataset	**Number of Participants**	**Number of WSIs**	**Number of tiles**
Training	10	36	2407
Internal Hold-out	2	4	551
UCLA External Test	1	2	573

Supplementary Table 6: Summary of datasets for blood vessel detection training, internal hold-out, and external testing

Supplementary Figure 1

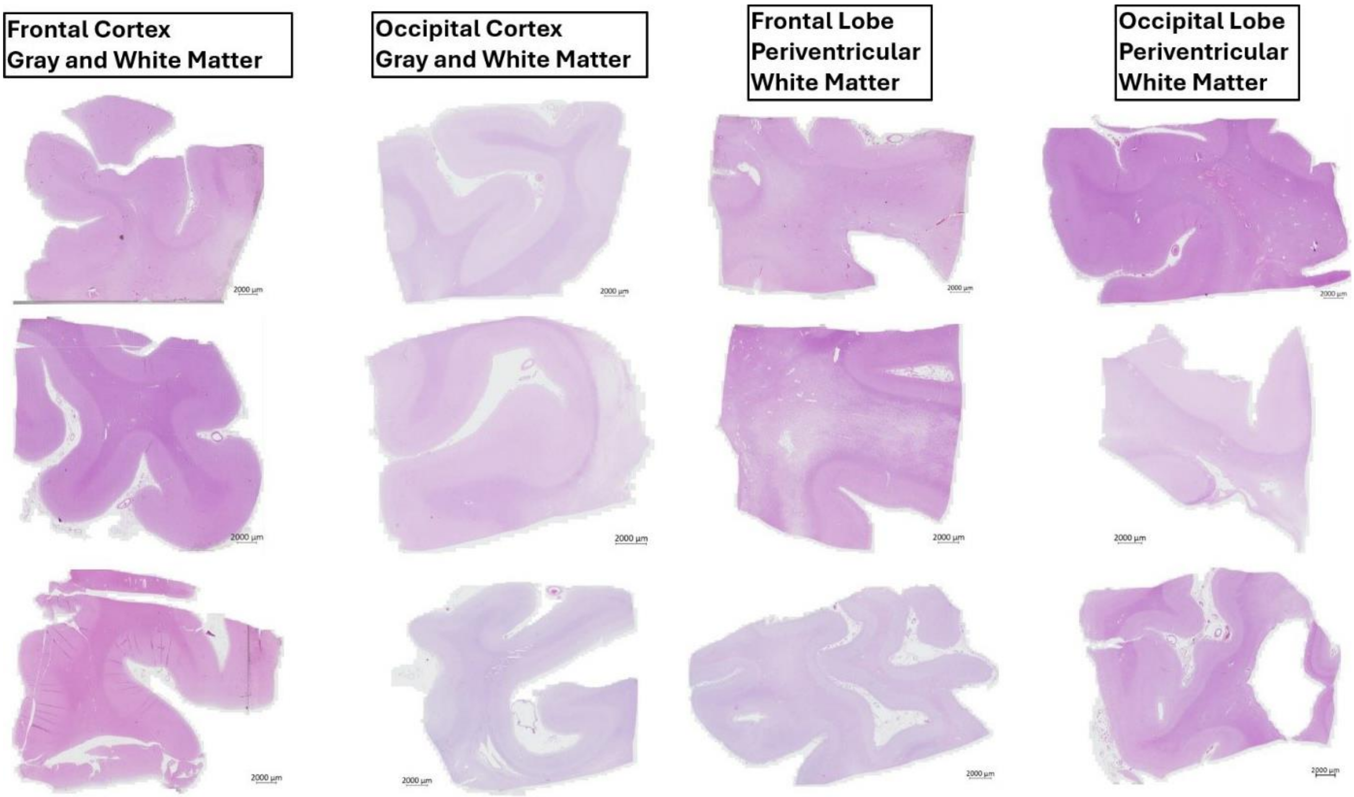

Supplementary Figure 1: Example WSIs of samples from cortical and/or periventricular white matter regions of the frontal and occipital lobes. Further details for each case are in **Supplementary Table 1**.

Copyright: © 2025 The author(s). This is an open access article distributed under the terms of the Creative Commons Attribution 4.0 International License (https://creativecommons.org/licenses/by/4.0/), which permits unrestricted use, distribution, and reproduction in any medium, provided the original author and source are credited, a link to the Creative Commons license is provided, and any changes are indicated. The Creative Commons Public Domain Dedication waiver (https://creativecommons.org/publicdomain/zero/1.0/) applies to the data made available in this article, unless otherwise stated.

Supplementary Figure 2

$$C = \frac{\sum_{i=1}^{n} s_i}{n}$$

Supplementary Figure 2: Equation for calculating a confidence score from segmentation output of the vessel detection model

C = confidence score

s_i = softmax probability for vessel class for each pixel where the softmax probability for the vessel is greater than that for the background

n = total number of pixels where the softmax probability for the vessel is greater than that for the background

Supplementary Figure 3

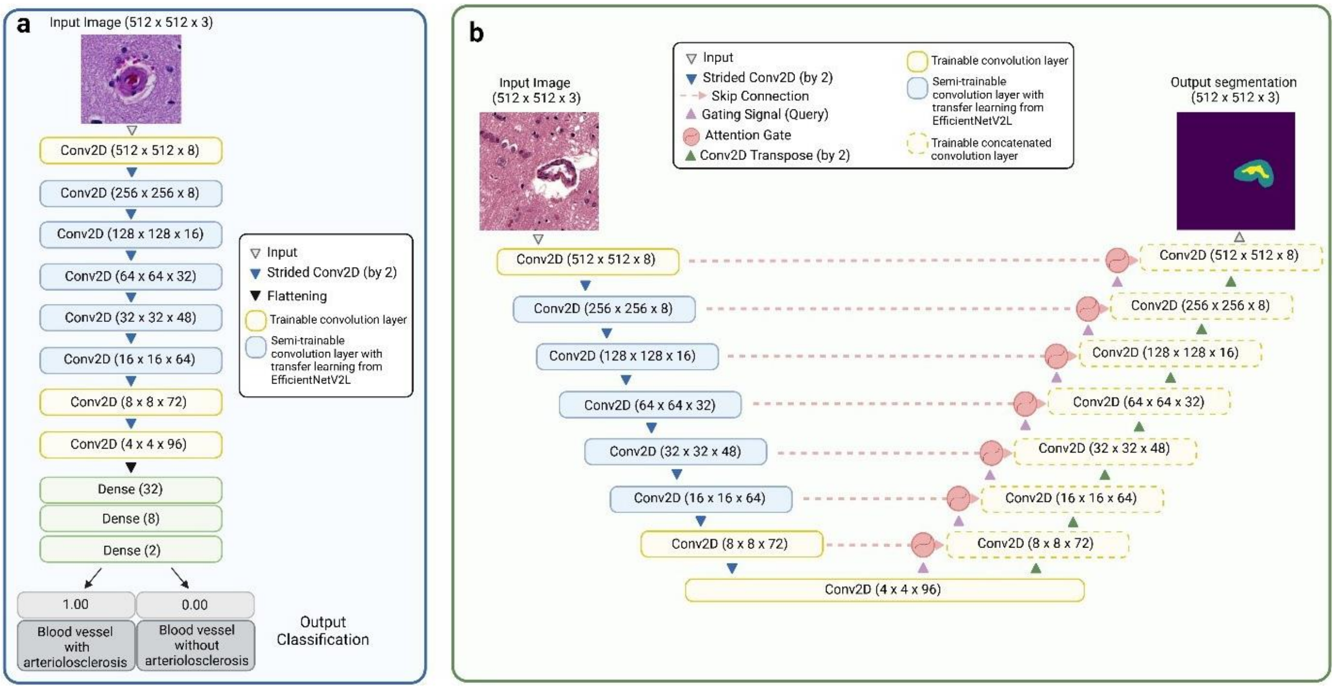

Supplementary Figure 3: Detailed architecture of the classification and segmentation ML models.

"Conv2D" operations consisted of separable 2D convolutions with kernel size = (3,3), stride = 1, and padding = 1, followed by batch normalization and Leaky ReLU. For subsampling, we utilized separable 2D convolutions with kernel size = (3, 3), stride = 2, and padding = 1. (**a**) The arteriolosclerosis classification model comprised an EfficientNetV2L[33] backbone with five semi-trainable convolution layers followed by two fully trainable convolution layers and three dense fully connected layers. The semi-trainable Conv2D layers involved concatenating the subsampling of the previous layer and an EfficientNet2VL layer with frozen parameters, followed by a "Conv2D" operation.The model contained 11 layers with 118,123,101 total parameters, 95,789,413 trainable parameters, and 22,333,688 untrainable parameters. (**b**) The blood vessel detection and arteriolosclerotic vessel segmentation models comprised an Attention U-net architecture[32] with an encoder composed of an EfficientNetV2L[33] backbone with five semi-trainable convolution layers followed by two fully trainable convolution layers and a decoder composed of seven trainable convolution layers generated by concatenating the 2D transpose convolution of the previous layer with kernel size = (3, 3), stride = 2, and padding = 1 and an attention gate[32] that filters features propagated from the skip connections. The model contained 118,409,937 total parameters, 96,074,899 trainable parameters, and 22,335,038 untrainable parameters.

Copyright: © 2025 The author(s). This is an open access article distributed under the terms of the Creative Commons Attribution 4.0 International License (https://creativecommons.org/licenses/by/4.0/), which permits unrestricted use, distribution, and reproduction in any medium, provided the original author and source are credited, a link to the Creative Commons license is provided, and any changes are indicated. The Creative Commons Public Domain Dedication waiver (https://creativecommons.org/publicdomain/zero/1.0/) applies to the data made available in this article, unless otherwise stated.
