# Supplementary material for: Applying machine learning to assist in the morphometric assessment of brain arteriolosclerosis through automation
Source: Free Neuropathol. 2025 Jun 2;6:12. doi: 10.17879/freeneuropathology-2025-6387 (PMC12159543; doi:10.17879/freeneuropathology-2025-6387)
Supplement: Supplementary file 4 [file freeneuropathol-06-12-6387-s4.docx]

**Arteriolosclerosis Annotation Protocol**

Create subfolder in each ID_XXX: ID_XXX_Masks, ID_XXX_Negative, ID_XXX_Positive ( create ID_XXX_Maybe, ID_XXX_Repeats, and ID_XXX_CannotAnnotate if needed)

Subfolder definition

- Masks: put the annotated masks here (instructions in the next section)
- Negative: not arteriolosclerosis
- Positive: arteriolosclerosis and able to annotate
- Maybe: not sure whether it’s positive/negative
- Repeats: put repeated tiles here without determining if they’re positive/negative
- CannotAnnotate: possible arteriolosclerosis but not able to annotate because of too small magnification or incomplete view of the vessel
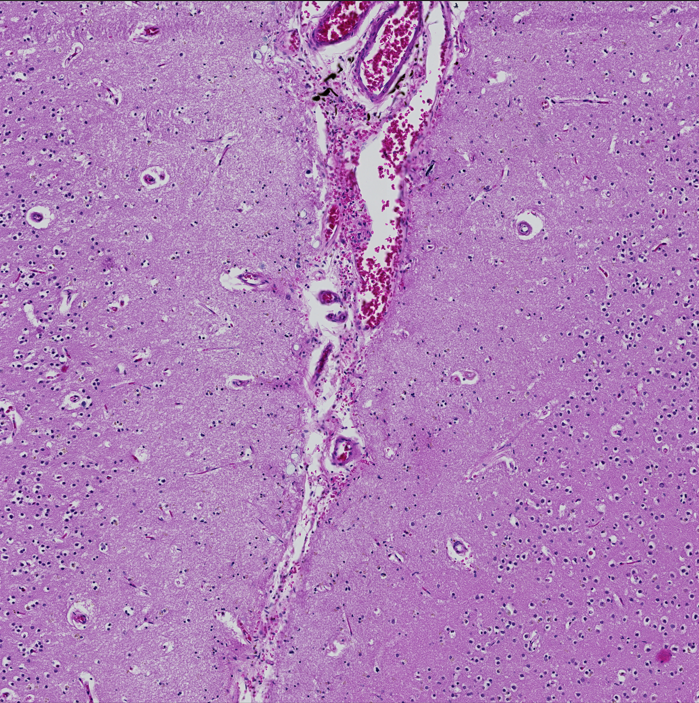


**Positive arteriolosclerosis**

- Cross-section rather than longitudinal
- Need to see the lumen clearly (or it’s hard to train the computer model)
- Need to be in white matter only, not in gray matter or leptomeninges

|  | Thickening of the media | Decreased lumen size | Loss of smooth muscle cells |
| --- | --- | --- | --- |
| None |  |  |  |
| Mild | ✔ |  |  |
| Moderate | ✔ | ✔ | ✔ |
| Severe | ✔ | ✔ | ✔ (complete loss) |

Reference:

[Vascular cognitive impairment neuropathology guidelines (VCING): the contribution of cerebrovascular pathology to cognitive impairment](https://academic.oup.com/brain/article/139/11/2957/2422120?login=true) (Skrobot et al, 2016)

graphs from [Brain Arteriolosclerosis](https://www.ncbi.nlm.nih.gov/pmc/articles/PMC8503820/) (Blevins et al, 2021)


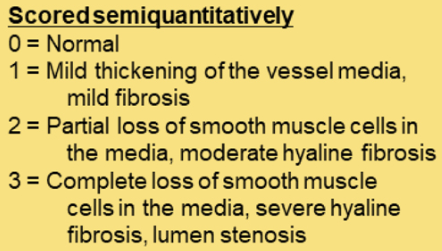

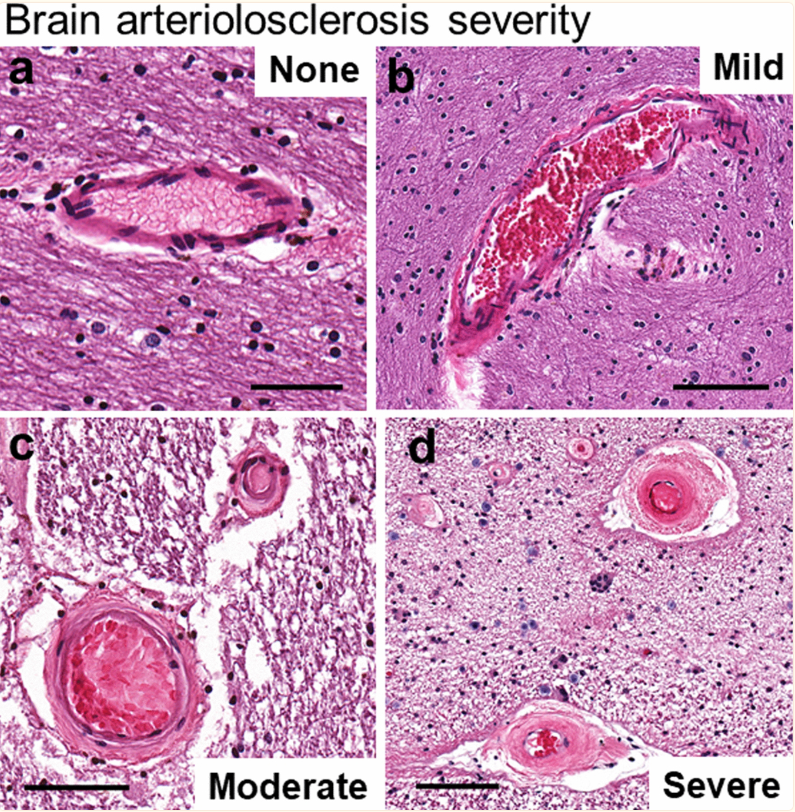


**How to annotate**

1. Open Image J (Fiji)
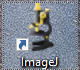

2. Copy the file name of the positive tile > drag the file to Image J window (or import via File > Open)
3. Double click the oval shape brush > check “Enable selection brush” > normally set 50 pixels for vessel border and 25 pixels for lumen border (can adjust based on different tiles)
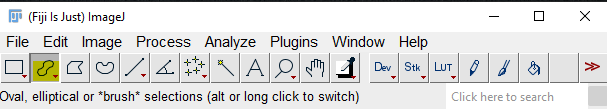

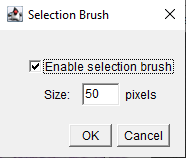

4. Draw the vessel border by holding left click
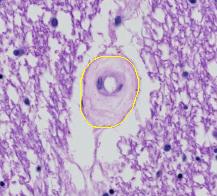

   1. If 2 positive vessels: shift and hold left click to draw
   2. To erase: draw from outside to trim the border
5. Click Edit > Selection > Create Mask
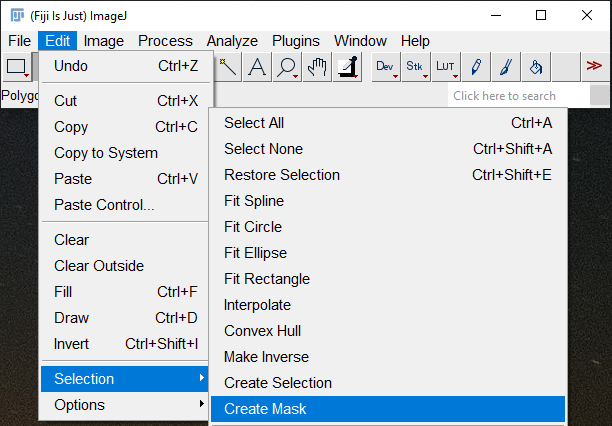

6. You should get the vessel area as white and the background as black as below. If not, click Edit > Invert (or Ctrl+Shift+I)
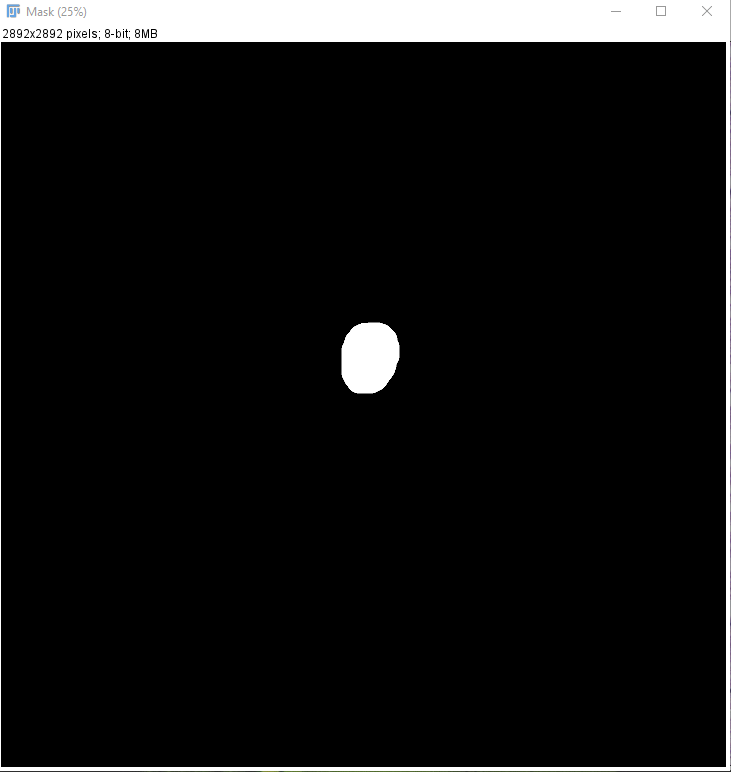

7. Save the mask by clicking File > Save As > PNG
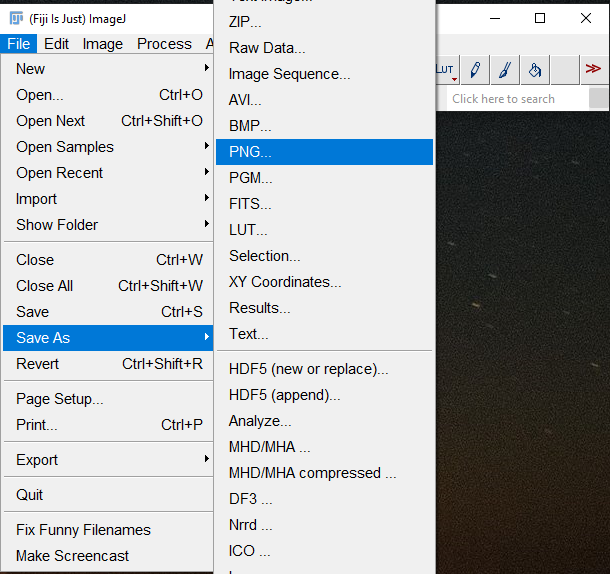

8. Paste the file name that you copied in Step 2 in front of the Mask.png (make sure to save the masks in the subfolder ID_XXX_Masks)
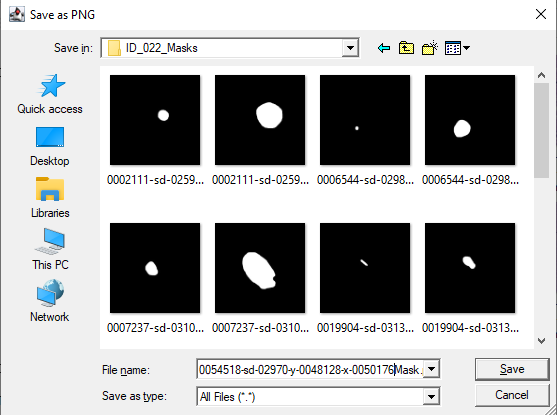

9. Close the mask window and go back to annotate the lumen border
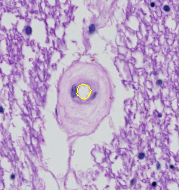

10. Create a mask the same as Step 5-7
11. Paste the file name that you copied in Step 2 and put L before Mask.png to indicate the lumen mask
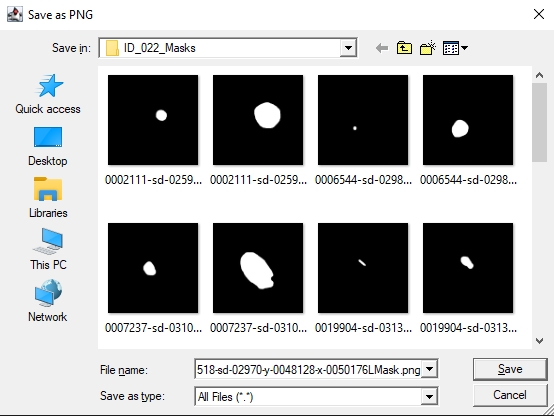

12. Repeat the steps above for the next tile
