# Supplementary material for: Applying machine learning to assist in the morphometric assessment of brain arteriolosclerosis through automation
Source: Free Neuropathol. 2025 Jun 2;6:12. doi: 10.17879/freeneuropathology-2025-6387 (PMC12159543; doi:10.17879/freeneuropathology-2025-6387)
Supplement: Supplementary file 5 [file freeneuropathol-06-12-6387-s5.docx]

To evaluate the two candidate definitions and methods for calculating vessel wall thickness, we created a test set (n = 62) by selecting images (Supplementary Figure 4) meeting the following inclusion criteria: (1) the image contained only one single vessel, (2) segmentation output showed uninterrupted circumferential labeling of the vessel wall, and (3) segmentation output does not show false-positive satellite lumens and only contains one accurately segmented lumen. A senior neuropathologist (WY) manually measured the vessel wall thickness for each image using ImageJ.^29^ For these manual measurements, blood vessel wall thickness was defined as the distance between the inner boundary of the vessel endothelium and the outer boundary of the tunica adventitia along a line perpendicular to the wall’s “backbone” or minimum skeleton – the arc equidistant from the outer and inner wall boundaries, which equates to the sum of the widths of the endothelium, tunica intima, tunica media, and tunica adventitia.^37^ The neuropathologist was asked to complete four vessel wall thickness measurements per image (Supplementary Figure 5).


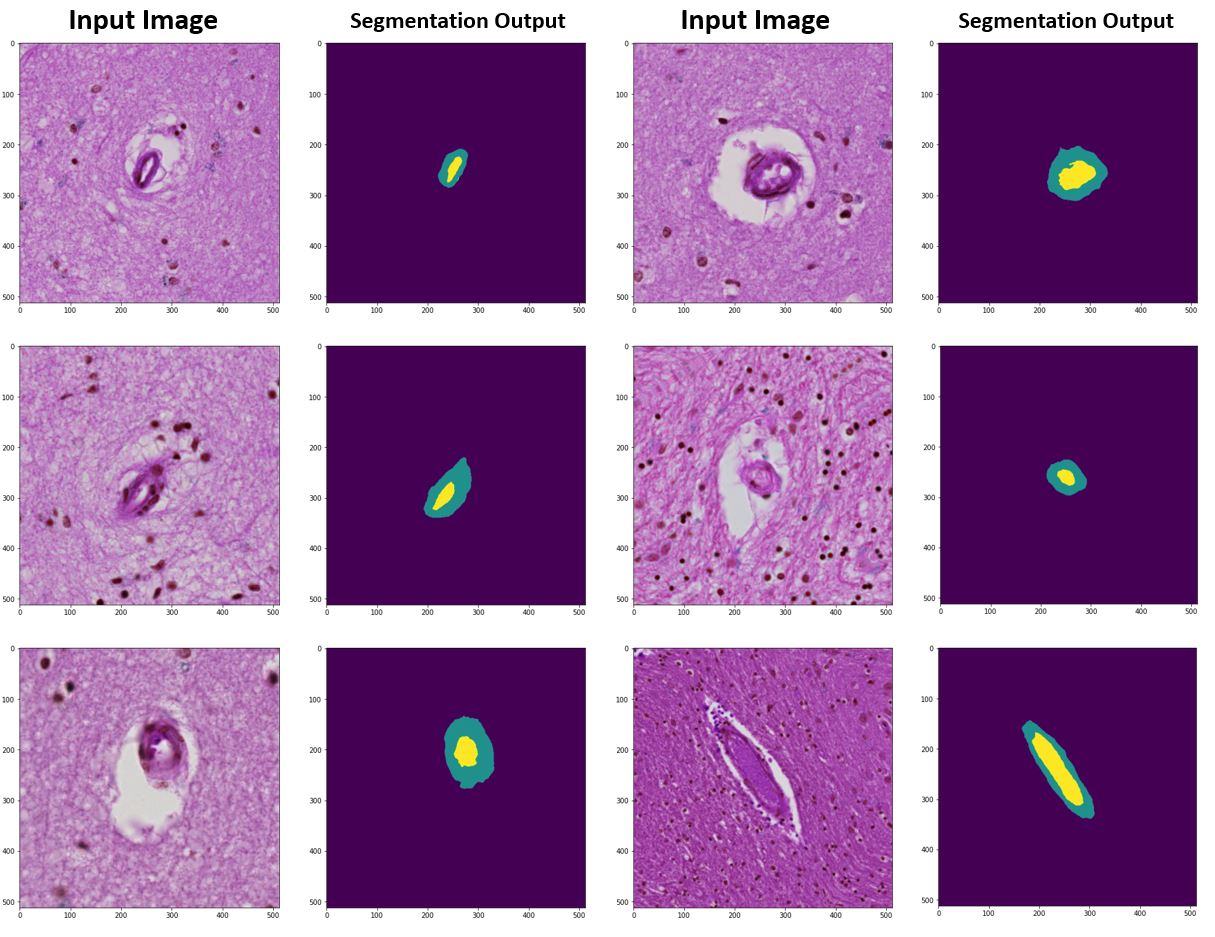


**Supplementary Figure 4: Example input image and segmentation output pairs in test set used to evaluate the two candidate definitions of vessel wall thickness.** The inclusion criteria for each image were: (1) the image contained only one single vessel, (2) segmentation output showed uninterrupted circumferential labeling of the vessel wall, and (3) segmentation output does not show false-positive satellite lumens and only contains one accurately segmented lumen. The test set included 62 total image and segmentation output pairs.


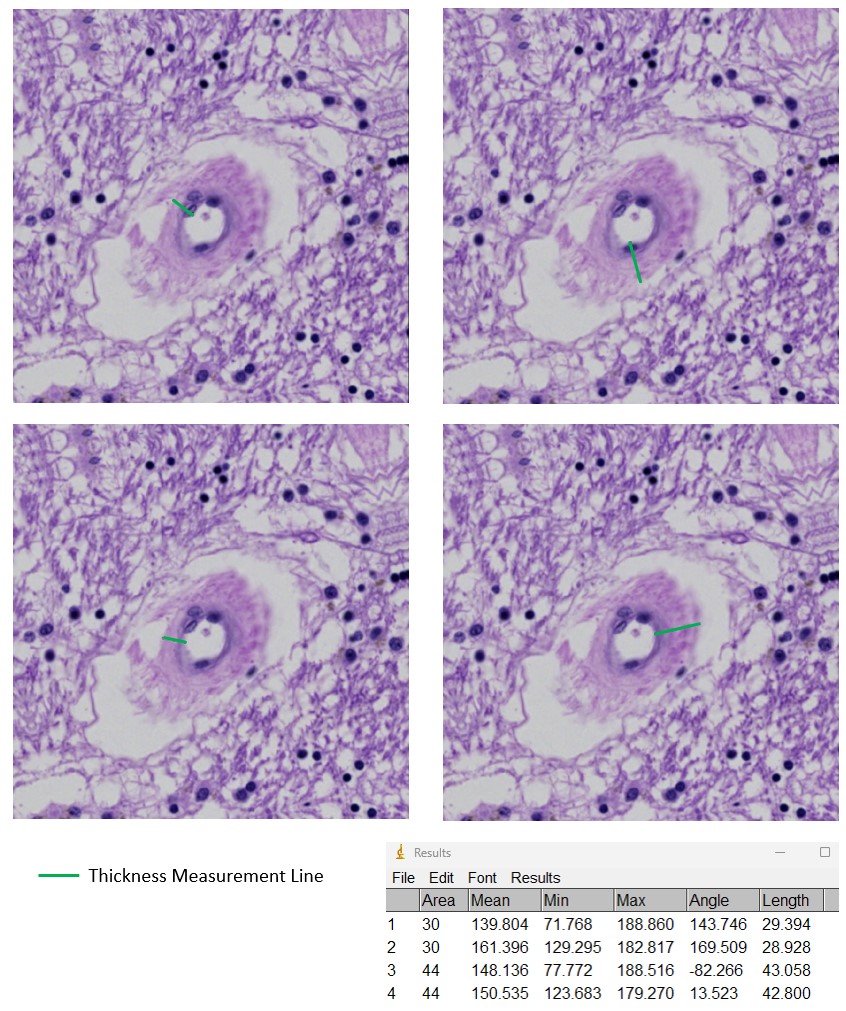


**Supplementary Figure 5: Example manual vessel wall thickness measurements for one image within the SITE test set.** Four manual measurements were made per image by a neuropathologist (WY). The test set contained 62 total images and segmentation outputs.
