# Supplementary material for: Applying machine learning to assist in the morphometric assessment of brain arteriolosclerosis through automation
Source: Free Neuropathol. 2025 Jun 2;6:12. doi: 10.17879/freeneuropathology-2025-6387 (PMC12159543; doi:10.17879/freeneuropathology-2025-6387)

**Arteriolosclerosis classification 3-fold cross validation model hyperparameters**

- batch_size = 30
- epochs = 50
- learning_rate = 1e-3
- learning_ratio = 0.99
- loss = tf.keras.losses.SparseCategoricalCrossentropy(from_logits=True)
- Training time: about 129 hours
- Inference time: about 12 hours

**Blood vessel detection and arteriolosclerotic vessel segmentation model hyperparameters**

- batch_size = 30
- epochs = 50
- learning_rate = 1e-2/5
- learning_ratio = 0.99
- loss = jerry_losses.focal_dice_like_loss_multiclass_weighted
- Training time: about 54 hours
- Inference time: about 8 hours

**jerry_losses.focal_dice_like_loss_multiclass_weighted**


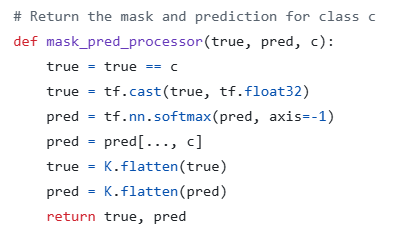


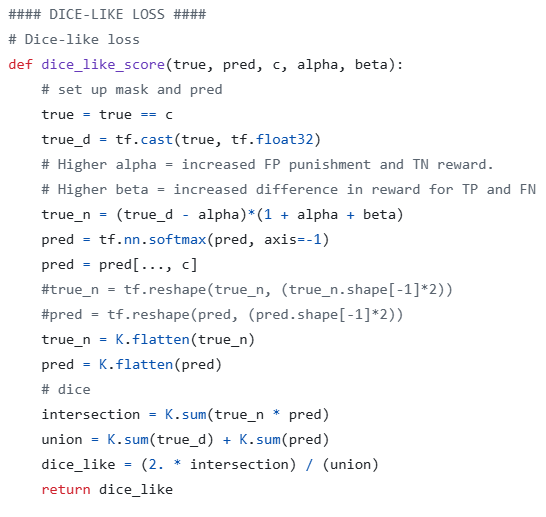


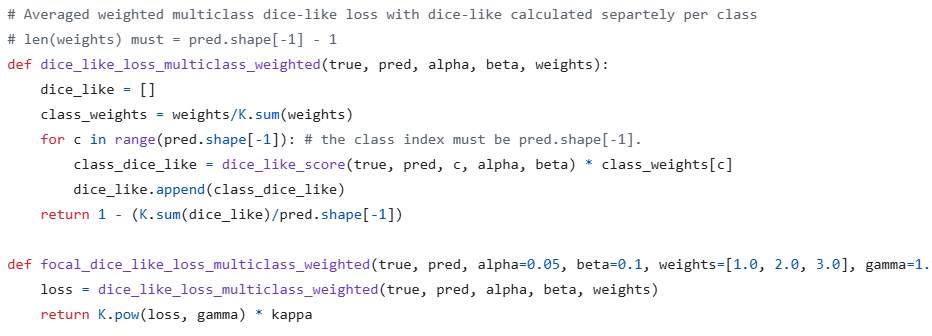

Supplement: Supplementary file 6 [file freeneuropathol-06-12-6387-s6.docx]
